# Oligomerization and exocyst coupling underlie Spa2-mediated focusing of polarized growth in fission yeast

**DOI:** 10.1242/jcs.264071

**Published:** 2025-09-11

**Authors:** Liping Ren, Alaina H. Willet, Kathleen L. Gould

**Affiliations:** Department of Cell and Developmental Biology, Vanderbilt University School of Medicine, Nashville, TN 37232, USA

**Keywords:** Polarized growth, Spa2, Exocyst complex, Fission yeast, Ypt2/Sec4, GTPase, RabGAP, Gyp3

## Abstract

Polarized cell growth in fungi requires the spatial restriction of exocytosis to discrete cortical domains. Defined by a characteristic domain architecture, the evolutionarily conserved scaffold protein Spa2 localizes to sites of polarized growth in fungi and has been implicated in morphogenic processes including hyphal extension in filamentous fungi and budding yeast mating. *Schizosaccharomyces pombe* is a well-studied and powerful model organism for elucidating mechanisms of polarized growth. However, identifying a role for Spa2 in *S. pombe* morphogenesis has been elusive, highlighting a gap in defining a broadly conserved Spa2 function. Here, we undertook a comprehensive and comparative dissection of the targeting mechanisms, interactome and function of Spa2 in *S. pombe*. We find that all of the conserved domains in Spa2 influence Spa2 localization to sites of polarized growth in an exocyst-dependent and largely cytoskeleton-independent manner. At cell tips, stable complexes of oligomerized Spa2 contribute to constraining the growth zone, in part by delivering the Rab GTPase-activating protein for the Sec4 homolog Ypt2. Despite species-specific wiring of Spa2 protein networks, our results underscore an evolutionarily conserved role for Spa2 in sharpening the spatial focus of polarized growth.

## Teaser

**Highlighted Article:** The Spa2 protein acts in fission yeast as a stable oligomeric scaffold to focus the zone of polarized growth, thereby contributing to the morphology of the organism.

## INTRODUCTION

A capacity for polarization is commonly observed among both eukaryotes and prokaryotes. In a single-celled context, cellular polarization governs important morphological processes such as directed growth, cell motility and fate specification. One factor linked to cell polarization in multiple yeast and fungal species including *Schizosaccharomyces pombe* (Ren et al., 2015)*, Saccharomyces cerevisiae* (Snyder, 1989), *Aspergillus nidulans* (Virag and Harris, 2006), *Ashbya gossypii* (Knechtle et al., 2003), *Ustilago maydis* (Carbó and Pérez-Martín, 2008), *Neurospora crassa* (Araujo-Palomares et al., 2009) and *Candida albicans* (Zheng et al., 2003) is Spa2. In *S. cerevisiae*, it has been well established that Spa2 serves as a scaffold within a multi-protein network, termed the polarisome, that promotes F-actin polymerization at cell tips and proper morphology (reviewed in Xie and Miao, 2021). The *S. cerevisiae* polarisome also contains Pea2 and the F-actin polymerization regulators Bud6, Aip5 and formin Bni1 (Fujiwara et al., 1998; Gehrung and Snyder, 1990; Glomb et al., 2019; Sheu et al., 1998; Xie et al., 2019). Type V myosin and Epo1 (an endoplasmic reticulum membrane protein) are also linked to Spa2 via interactions with Pea2 (Chao et al., 2014; Dünkler et al., 2021; Neller et al., 2015). Homologs of Spa2, termed GIT1 and GIT2 (collectively, GIT proteins), also exist in human and function as Arf-specific GTPase-activating proteins (GAPs) in multiple biological processes (reviewed in Zhou et al., 2016).

Spa2 proteins in different species of yeast and filamentous fungi have different sizes, domain organization and interactomes, as well as variable significance to morphogenesis (Fig. S1) (Xie and Miao, 2021). Unlike in *S. cerevisiae*, deletion of *spa2* orthologs in *U. maydis* (Carbó and Pérez-Martín, 2008), *A. gossypii* (Knechtle et al., 2003) and *C. albicans* (Zheng et al., 2003) does not significantly affect F-actin filament organization at growing cell tips, and Spa2 proteins persist at the poles of *U. maydis* and *A. gossypii* cells when the F-actin or microtubule cytoskeletons are disrupted (Carbó and Pérez-Martín, 2008; Köhli et al., 2008). Further, sequence orthologs of *S. cerevisiae* Pea2 do not exist in *Schizosaccharomyces* yeast species or most filamentous fungi (Fig. S1B), underscoring the variability of Spa2 interactomes. The fission yeast *S. pombe* is a powerful and popular model organism for studying mechanisms by which polarization is established, maintained and modulated (Chiou et al., 2017; Martin and Arkowitz, 2014). *S. pombe* cells are shaped as cylinders with rounded ends, and their growth is limited to cell tips. Immediately after medial cell division, *S. pombe* cells elongate at only the ends inherited from mother cells. Then, at a later point in G2 known as new end take-off (NETO), cells transition to a bipolar growth mode and also extend from the new ends established by cell division (Mitchison and Nurse, 1985). *S. pombe* Spa2 was identified as an interactor of SH3 domains from the cytokinesis F-BAR proteins Cdc15 and Imp2 (Ren et al., 2015). Confoundingly, in contrast to deletion mutants of Spa2 proteins in many organisms, *S. pombe spa2*Δ cells are not observed to have noticeable defects in polarized vegetative growth, NETO, formation of mating projections, width of mating projections, or mating efficiency, although a minor defect in mating projection targeting has been noted (Dudin et al., 2017; Ren et al., 2015).

Despite the variable interactomes and contributions to morphogenesis in different organisms, all Spa2 proteins localize strongly to sites of polarized growth and serve as useful markers of cell polarization (Xie and Miao, 2021). This suggests that there is a common yet undefined mechanism by which all Spa2 proteins target the cell cortex at polarization sites. Spa2 and GIT proteins have also been reported to oligomerize (Premont et al., 2004; Zheng et al., 2020) and to undergo liquid–liquid phase separation (Xie et al., 2019; Zhu et al., 2020), although the significance of this to Spa2 protein function *in vivo* is not clear. To advance our understanding of what governs polarized localization of Spa2 and to provide clues as to a core evolutionarily conserved function for *S. pombe* Spa2, we comprehensively characterized the motifs and domains within *S. pombe* Spa2 that influence its localization and compared its overall organization and interactome with Spa2 proteins from other organisms. We also examined the oligomerization capacity of Spa2 and its significance, and we identified the exocyst as being essential for Spa2 localization to sites of polarized growth. Lastly, we re-examined the role of Spa2 in *S. pombe* morphology and found that Spa2 is required to focus the zone of polarized vegetative growth. We postulate that concentrating the area of exocytosis is an evolutionarily conserved function of Spa2 proteins.

## RESULTS

### Multiple domains and motifs contribute to proper Spa2 intracellular targeting

**Fig. 1.**
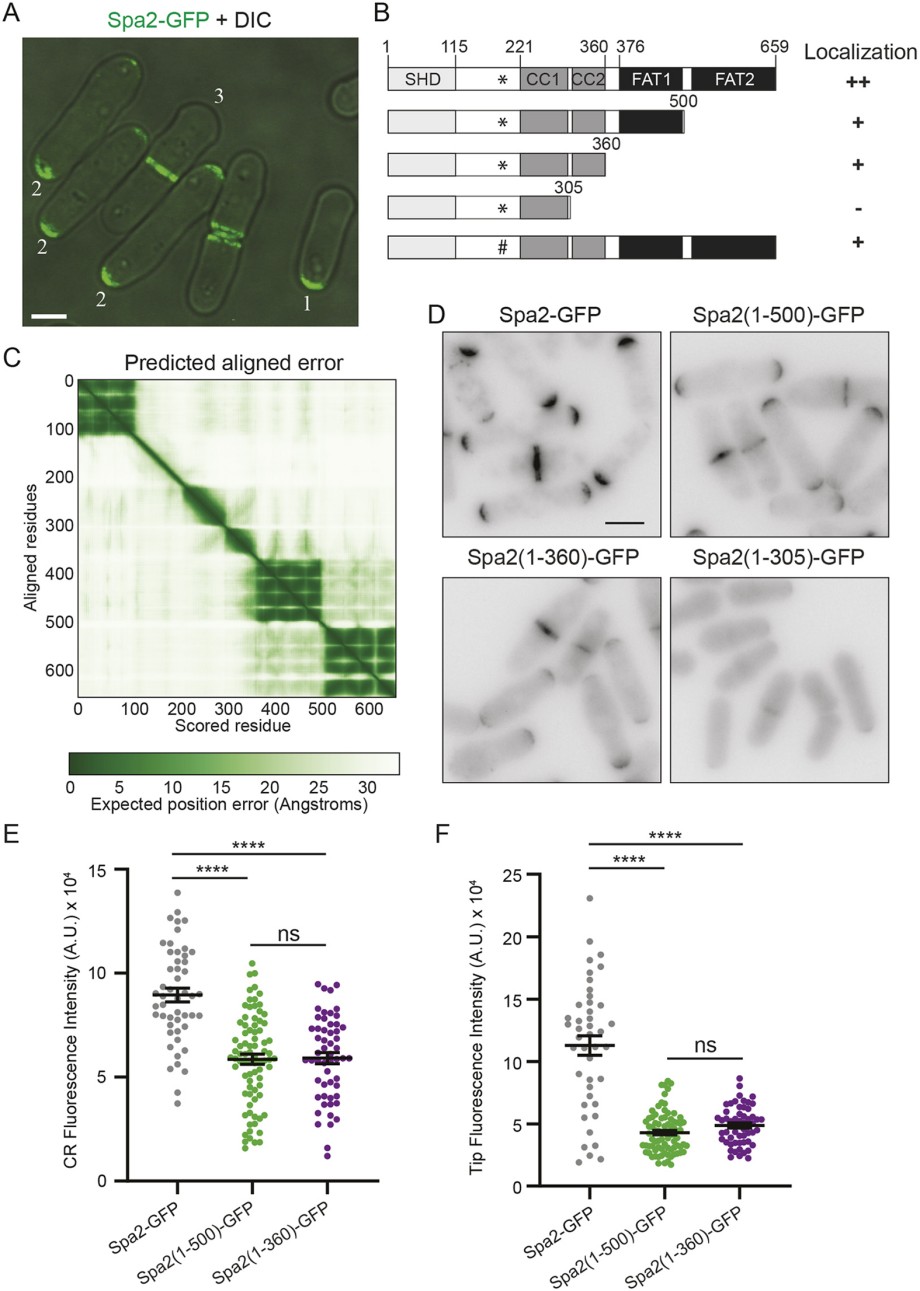
**C-terminal sequences contribute to Spa2 localization.** (A) Live-cell image of merged Spa2–GFP and DIC signals, with cell types defined as follows: (1) Spa2 at one tip; (2) Spa2 at two tips; (3) Spa2 at the CR. Scale bar: 3 µm. (B) Left, a schematic diagram of Spa2 drawn to scale with the Spa2 homology domain (SHD), coiled-coil (CC) domains and focal adhesion targeting (FAT) domains indicated. The PXXP motif (residues 191–194), which binds the SH3 domains of Cdc15 and Imp2 (Ren et al., 2015), is marked with an asterisk. The P191A mutation is indicated with #. Right, summary of the localization of the indicated Spa2 truncation proteins from D and Ren et al. (2015). (C) AF database (https://alphafold.com/)-generated predicted aligned error plot for Spa2 (Jumper et al., 2021; Varadi et al., 2022). (D) Live-cell images of *spa2–GFP*, *spa2(1–500)–GFP*, *spa2(1–360)–GFP* and *spa2(1–305)–GFP* cells. Scale bar: 5 µm. (E,F) Quantitation of fluorescence intensity of Spa2–GFP at cell tips (E) or CRs (F). Data are from two separate experiments with *n*≥42 for each. Error bars represent mean±s.e.m. ****P<0.0001; ns, not significant (one-way ANOVA with Tukey's post-test). Spa2(1–500)–GFP versus Spa2(1–360)–GFP at CR *P*=0.98 and at cell tips *P*=0.47. A.U., arbitrary units.

To gain insight into how *S. pombe* Spa2 localizes to tips and the division site (Fig. 1A), we first examined the organization of the protein. *S. pombe* Spa2 contains several recognizable sequence elements, including the hallmark Spa2 homology domain (SHD) at the N terminus and a predicted coiled-coil (CC) domain (CC1; Fig. 1B). It also contains a PXXP motif between the SHD and CC1, which binds the SH3 domains of cytokinetic F-BAR proteins Cdc15 and Imp2 and contributes modestly to its localization (Ren et al., 2015) (Fig. 1B). In addition, when examined using AlphaFold (AF) (Jumper et al., 2021; Varadi et al., 2022), other domains of predicted structure emerged, as visualized by the predicted aligned error plot (Fig. 1C). These included a second α-helical segment (CC2) and two focal adhesion targeting (FAT)-like domains (FAT1 and FAT2; Fig. 1B,C) (Holm, 2022; van Kempen et al., 2024). A comparison of domain organization showed that the SHD (also called SHD-1) is found in all Spa2 (Fig. S1A) and GIT proteins; the SHD structure is also conserved from fungi to human (Zheng et al., 2020; Zhu et al., 2020). All Spa2 proteins also have a CC domain. Two FAT domains (also called SHD-II and SHD-V), each comprising a four-helix bundle, are present in all Spa2 proteins and are often separated by significant stretches of amino acids predicted to be unstructured. It is also evident that *S. pombe* Spa2 is the shortest protein among the homologs (Fig. S1A).

To determine which of the motifs and/or domains contribute to *S. pombe* Spa2 localization, we generated a series of C-terminal truncations by inserting GFP at selected amino acids (Fig. 1B). Although the C-terminal truncation mutants Spa2(1–500) and Spa2(1–360) localized to cell tips and septa like full-length Spa2, they were both reduced in intensity by 3-fold and 1.5-fold relative to full-length Spa2 at tips and the division site, respectively (Fig. 1D–F; Fig. S2A,B). However, Spa2(1–500)–GFP and Spa2(1–360)–GFP intensities were not different from each other (Fig. 1D–F; Fig. S2C,D). Truncating the C terminus further to the predicted CC1 region (amino acid 305) abolished plasma membrane (PM) localization (Fig. 1D). Despite reduced cortical targeting, there was no change in abundance of the truncated proteins, relative to full-length Spa2, as determined by whole-cell fluorescence intensity and quantitative immunoblotting (Fig. S2D,E). Thus, the terminal FAT domain and CC2 contribute to normal Spa2 localization.

**Fig. 2.**
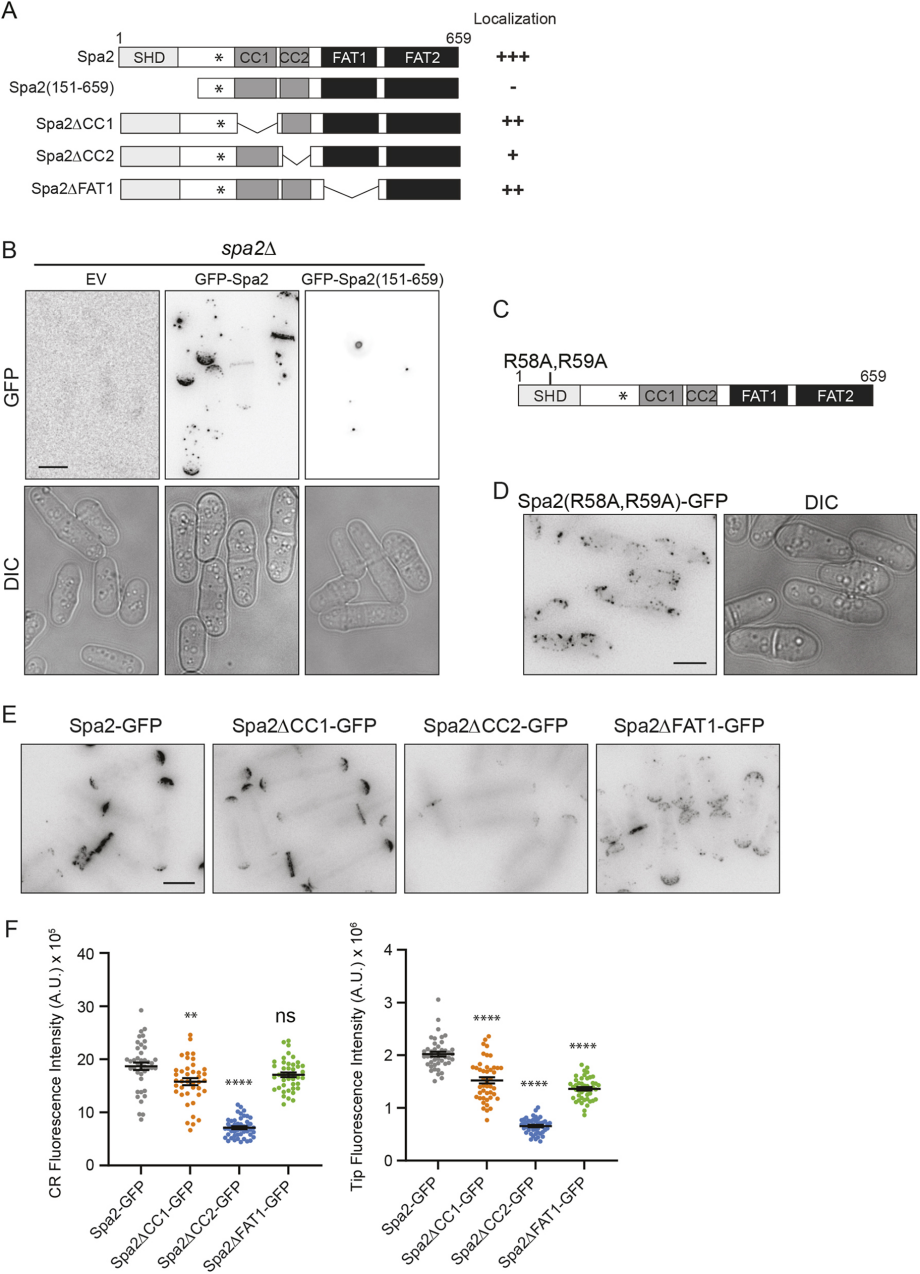
**The SHD is essential for Spa2 localization.** (A,C) Schematic diagrams of the indicated Spa2 mutants annotated with the domains defined in Fig. 1B. The ability of Spa2 mutants to localize like wild-type Spa2 is indicated on the right of A. (B) Representative live-cell images of the indicated GFP–Spa2 fusion proteins expressed exogenously from the depressed *nmt81* promoter in *spa2*Δ cells. Images are representative of two experiments. EV, empty vector. (D,E) Representative live-cell images of the indicated Spa2 proteins produced from the endogenous *spa2* locus. Images in D are representative of two experiments. (F) Quantitation of fluorescence intensity of the indicated Spa2 proteins at CRs (left) or cell tips (right). Data are from three separate experiments with *n*≥38 for each. Error bars represent mean±s.e.m. ****P<0.0001; **P<0.01; ns, not significant (one-way ANOVA with Tukey's post-test). Spa2–GFP versus Spa2ΔFAT1–GFP at CR, *P*=0.15. A.U., arbitrary units. Scale bars: 5 µm.

To determine whether the SHD domain influenced Spa2 localization, we expressed GFP–Spa2 or GFP–Spa2(151–659) exogenously in *spa2*Δ cells. It is important to note that overexpression of Spa2 causes defects in morphology (Ren et al., 2015). Whereas full-length GFP–Spa2 localized to cell tips and septa, GFP–Spa2(151–659) did not, appearing instead as cytoplasmic puncta (Fig. 2A,B). Residues required for SHD-mediated protein interactions in the context of human GIT1 (Kim et al., 2003) are conserved in the Spa2 SHD (Zheng et al., 2020), and we replaced *spa2–GFP* with a mutant version in which sequences encoding two of the conserved SHD residues (R58 and R59) were mutated to encode alanines (Fig. 2C). Spa2(R58A, R59A)–GFP formed puncta and did not localize to the PM (Fig. 2D). Spa2(R58A, R59A)-GFP was also around three times less abundant than Spa2–GFP, as determined by quantitative immunoblotting (Fig. S2E). Thus, the SHD domain is critical for the proper level and localization of Spa2.

Lastly, we made internal deletion mutants of individual domains at the endogenous *spa2* locus and tagged them with GFP (Fig. 2A,E). We found that all Spa2 internal deletions had somewhat reduced whole-cell protein levels, so further quantifications in these strains were normalized to overall protein levels. Spa2 lacking CC1 was reduced in abundance at the cell division site by ∼12% and at cell tips by ∼25% compared to levels of wild-type Spa2 (Fig. 2E,F; Fig. S2F). Spa2 lacking CC2 was more significantly reduced in abundance at both the cell division site (∼60%) and cell tips (∼67%) (Fig. 2E,F; Fig. S2F). Spa2 lacking FAT1 did not have a change in cell division site localization but was decreased in abundance at cell tips by ∼32% compared to levels of wild-type Spa2 (Fig. 2E,F; Fig. S2F). We noticed additional differences when comparing Spa2ΔFAT1 to wild-type Spa2 including: (1) Spa2ΔFAT1 localized to tips and the division site simultaneously, whereas Spa2 was detected at tips or at the division site but not both; (2) Spa2ΔFAT1 appeared in internal puncta, which were especially apparent in the cell middle of interphase cells, and this was never observed for wild-type Spa2; and (3) Spa2ΔFAT1 cell tip localization appeared broader than that of Spa2 (Fig. 2E).

Taken together, our data indicate that in addition to the Spa2 PXXP motif, which interacts with the SH3 domains of F-BAR proteins Cdc15 and Imp2 (Ren et al., 2015), Spa2 localization is supported by the CC1 region (residues 221–305), CC2 region (residues 310–360) and both FAT domains, and requires the SHD.

### The Spa2 CC2 region interacts with Pos1

**Fig. 3.**
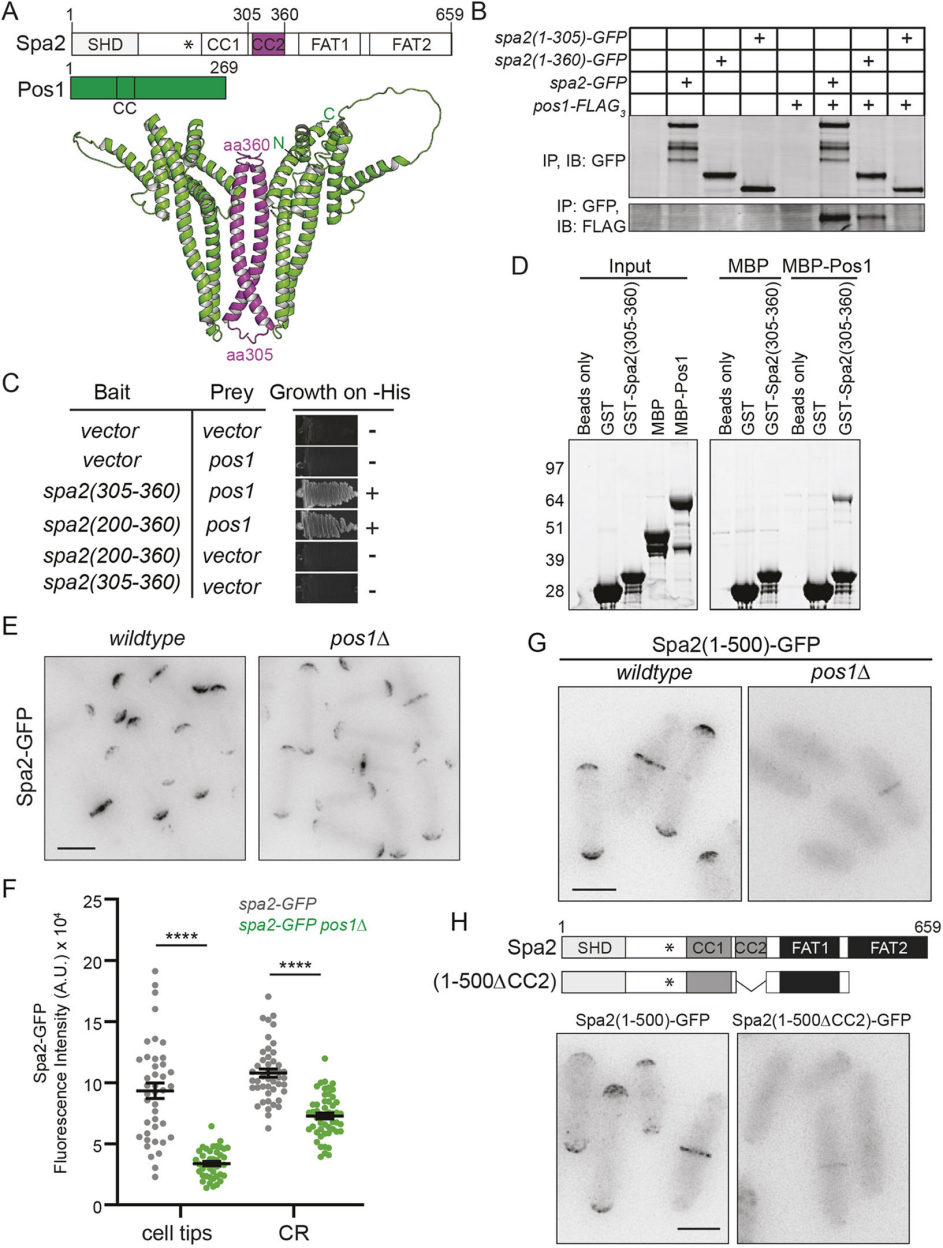
**Pos1 directly binds Spa2 and promotes Spa2 localization.** (A) Two copies of full-length Spa2 and two copies of full-length Pos1 were inputted to AF3 to predict a potential binding interface (Abramson et al., 2024). The protein schematics at the top indicate which section of each protein (magenta for Spa2 residues 305–360 and green for full-length Pos1) are included in the ribbon diagram below. The position of the N and C termini of each polypeptide in the model are indicated. The position of the PXXP motif is marked with an asterisk. aa, amino acid. (B) An anti-GFP (upper panel) and anti-FLAG (lower panel) immunoblot (IB) of anti-GFP immunoprecipitations (IP) from the indicated strains. (C) Yeast two-hybrid assay results using the indicated fragments of *spa2* tested for interactions with *pos1*. Growth on medium lacking histidine is shown, with ‘+’ indicating grown and ‘−’ indicating no growth. The majority of the yeast two-hybrid results presented in C and in Fig. S3C,E are derived from experiments that were performed at the same time, and some streaks of relevant negative controls have been duplicated in these panels. (D) *In vitro* binding assay with beads, bead-bound GST or bead-bound GST–Spa2(305–360) incubated with MBP or MBP–Pos1. Inputs (equal to the amount used for the binding reactions) are shown on the left. Binding reactions were washed and resolved with SDS-PAGE. Gels were stained with Coomassie Blue and imaged with an Odyssey CLx instrument. Molecular masses are indicated in kDa. (E) Representative live-cell images of *spa2–GFP* and *spa2–GFP pos1*Δ cells. (F) Quantitation of fluorescence intensity of Spa2-GFP at cell tips or CRs in the indicated genetic backgrounds. The graph represents data from two separate experiments with *n*≥41 for each strain and position. Error bars represent mean±s.e.m. ****P<0.0001 (unpaired, two-tailed Student's *t*-test). A.U., arbitrary units. (G) Representative live-cell images of Spa2(1–500)–GFP in wild-type and *pos1*Δ cells. (H) Representative live-cell images of Spa2(1–500)–GFP and Spa2(1–500ΔCC2)–GFP, alongside schematic diagrams showing the structure of Spa2(1–500ΔCC2). Asterisks mark the position of the PXXP motif. Data shown in B–D,G and H are representative of two experiments. Scale bars: 5 µm. All biochemical experiments were performed twice and all imaging experiments were performed three times.

Through an undefined region, *S. pombe* Spa2 binds stably to Pos1, a *Schizosaccharomyces*-specific protein (Ren et al., 2015; Rutherford et al., 2024) (Fig. S1B). Modeling of Spa2 and Pos1 using AlphaFold3 (AF3) (Abramson et al., 2024) predicted an interaction in which each of two Pos1 molecules bind to one side of a parallel dimer formed by Spa2 CC2 (Fig. 3A). In accord with this model, a *spa2* construct encoding residues 305–360 [*spa2(305–360)*] supported *pos1* interaction in a yeast two-hybrid assay (Fig. 3C) and recombinant GST–Spa2(305–360) bound MBP–Pos1 but not MBP *in vitro* (Fig. 3D). Further, we found that full-length Spa2–GFP and Spa2(1–360)–GFP co-immunoprecipitated FLAG-tagged Pos1; however, Spa2(1–305)–GFP did not (Fig. 3B). These results indicate that Pos1 binds the Spa2 CC2.

Given the role of CC2 in Spa2 localization determined above, we reasoned that the Spa2–Pos1 interaction might modulate Spa2 distribution. Although Spa2 localizes to its typical sites in *pos1*Δ cells (Ren et al., 2015), quantification revealed that less Spa2–GFP was present at tips and the division site of *pos1*Δ cells (43% and 80% of the levels seen in wild-type cells, respectively) (Fig. 3E,F) despite total levels of Spa2–GFP being unchanged, as determined by measuring whole-cell fluorescence intensity and immunoblotting (Figs S2E and S3A,B).

We next asked whether the loss of Spa2 localization exhibited by C-terminal truncation to residue 305 (see Fig. 1D) could be recapitulated by a combination of loss of the FAT2 domain together with loss of Pos1 binding. Indeed, Spa2 lacking FAT2 [Spa2(1–500)–GFP], which is normally only moderately reduced in membrane intensity (see Fig. 1D), was almost entirely cytosolic in *pos1*Δ cells (Fig. 3G). Further, Spa2 lacking both CC2 and FAT2 was also almost entirely cytoplasmic (Fig. 3H). These data are consistent with independent and possibly redundant contributions of Pos1 and the FAT2 domain to Spa2 localization.

### Spa2 and Pos1 self-associate

**Fig. 4.**
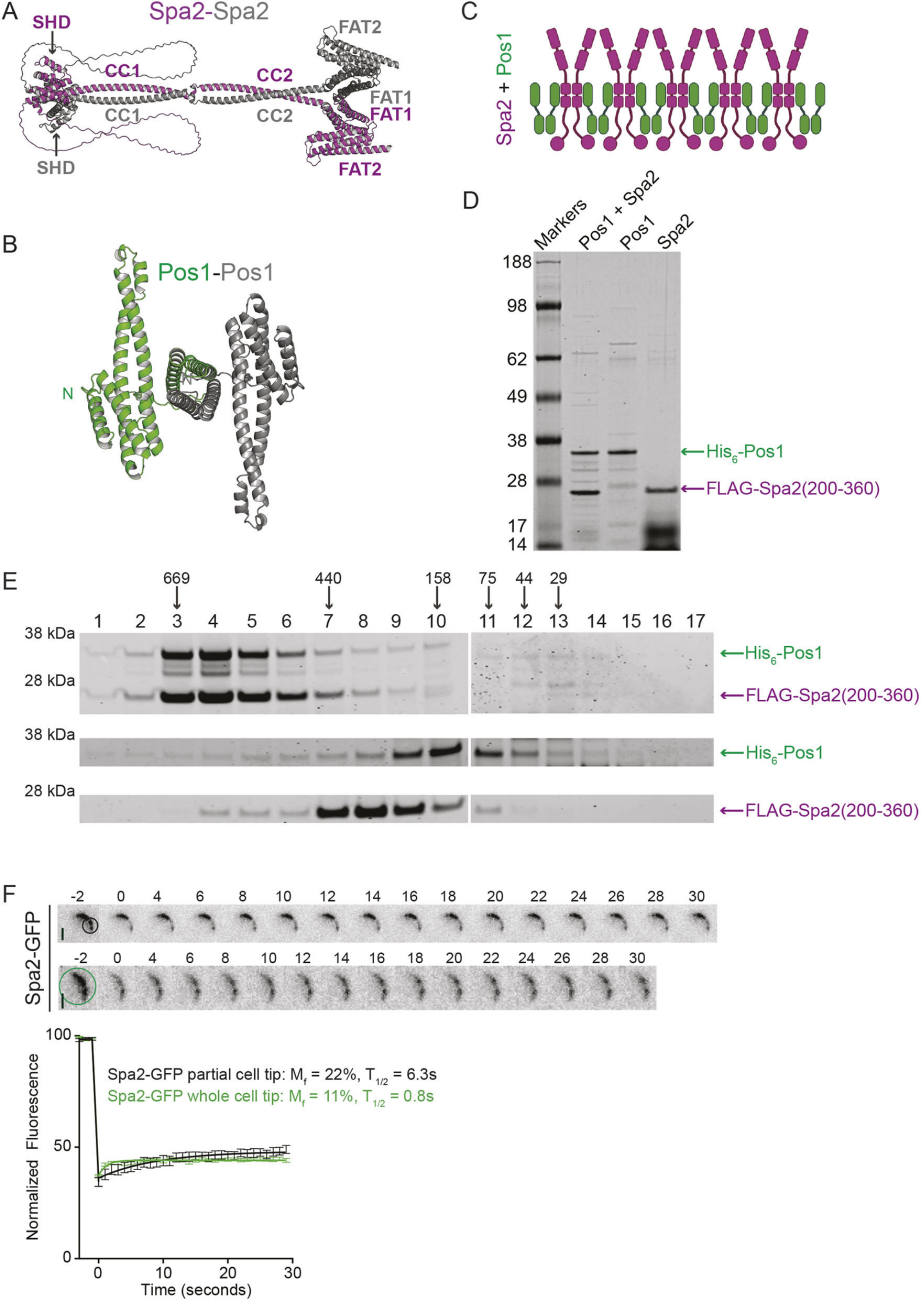
**Defining self-interaction domains of Spa2 and Pos1.** (A) AF3-generated model of a dimer of full-length Spa2. The SHD, CC1, CC2, FAT1 and FAT2 domains are indicated on the model. One monomer is magenta and the other monomer is gray. (B) AF3-generated model of a dimer of full-length Pos1. The N termini are indicated on the model. One monomer is green and the other monomer is gray. (C) A model of how Spa2–Pos1 might oligomerize. (D,E) Recombinantly purified His_6_–Pos1 and Spa2(200–360)–FLAG (Pos1+Spa2), His_6_–Pos1 alone (Pos1), or Spa2(200–360)–FLAG alone (Spa2) were analyzed by SDS-PAGE and Coomassie Blue staining (D). Each was then fractionated by gel filtration. A portion of each fraction was collected, and samples were resolved by SDS-PAGE and detected with Coomassie Blue staining (E). In D, molecular masses are shown in kDa. In E, the position of molecular size standards (in kDa) is indicated above the gels and fraction numbers. Data in D and E are representative of at least two experiments. (F) Top, images from representative live-cell time-lapse movies of *spa2–GFP* cells. Fluorescence of Spa2–GFP was photobleached including the entire cell tip or a section of the cell tip (as indicated by the circles) at time 0, and cells were imaged every 1 s. Every other time point is shown. Scale bars: 2 μm. Bottom, quantification of the fluorescence recovery after photobleaching of Spa2–GFP. Error bars represent mean±s.e.m. *n*=14 from three independent experiments. M_f_, mobile fraction; T_1/2_, half time of recovery.

In human GIT1, the CC domain supports self-association (Paris et al., 2003; Schlenker and Rittinger, 2009). Similarly, AF3 predicted that Spa2 CC1 and CC2 form a parallel dimer (Fig. 4A). Consistent with this model, two-hybrid analysis revealed that the Spa2–Spa2 interaction required both the putative CC1 domain and the Pos1-interacting CC2, and that the region containing CC1 and CC2 was necessary and sufficient for self-interaction (Fig. S3C). Further, Spa2–FLAG co-immunoprecipitated Spa2–GFP from a *spa2–FLAG spa2–GFP* diploid strain (Fig. S3D). Co-immunoprecipitation of Spa2–FLAG with Spa2–GFP was only modestly reduced in a diploid strain lacking Pos1, indicating that Pos1 does not significantly affect Spa2 self-association (Fig. S3D).

AF3 predicted that Pos1 also forms a dimer mediated by its last two C-terminal helices (Fig. 4B). In accord, Pos1 interacted with itself in a two-hybrid assay, and the last 69 amino acids were sufficient for self-association (Fig. S3E). Since the region of Pos1 interacting with Spa2 was distinct from its self-interaction motif, this raised the possibility that each subunit of dimeric Pos1 might bind a dimer of Spa2 to form an oligomerized complex larger than a tetramer (Fig. 4C). To test this idea, we used a dual expression system to co-produce His_6_-tagged Pos1 (33 kDa) with FLAG–Spa2(200–360) (19.5 kDa) recombinantly. The purified complex consisted of each protein in an approximate ratio of 1:1 (Fig. 4D). By size exclusion chromatography, a single major elution peak of the complex corresponding to ∼624 kDa was detected, indicative of extensive multimerization (Fig. 4E, top panels). When Pos1 and Spa2 were analyzed by themselves, they eluted from the column significantly later, with estimated molecular masses of 246 kDa and 305 kDa, respectively (Fig. 4E, middle and bottom panels). This might indicate that each protein forms multimers more complicated than dimers or that due to being rod shaped, they are retained longer on the column than if they had compact shapes. AF3 was used to investigate this question. When two, three or four copies of Spa2 and Pos1 were modeled, we found that the highest ranked predicted structures for both were the dimeric forms, as determined by the interface predicted template modeling (ipTM) score (Fig. S3F) (Cummins et al., 2022; Lin et al., 2025 preprint). Thus, we favor a model in which Spa2 and Pos1 each form dimers in the absence of the other. When combined, Spa2 and Pos1 have the capacity to form a large complex, and the polarized localization of Spa2 is likely facilitated by this mechanism.

Oligomers containing Spa2 have been reported to undergo liquid–liquid phase separation to form dynamic condensates *in vitro* (Xie et al., 2019; Zhu et al., 2020). In contrast, our biochemical results suggested that the Spa2–Pos1 complex is stable and of a discrete size. To test whether Spa2 complexes in cells were dynamic, consistent with a liquid-like condensate (Kenworthy, 2023; Taylor et al., 2019), fluorescence recovery after photobleaching (FRAP) analysis was used. After the entire cell tip or one half of a cell tip containing Spa2–GFP were bleached, we found that the mobile fractions were only 11% and 22%, respectively, and the total recovery times were 12.6 s and 1.6 s, respectively (Fig. 4F). These recovery parameters are very similar to those of GIT protein puncta *in vivo* (Zhu et al., 2020) and suggest that Spa2 complexes at the cell tip are quite stable.

### SHD interactions

**Fig. 5.**
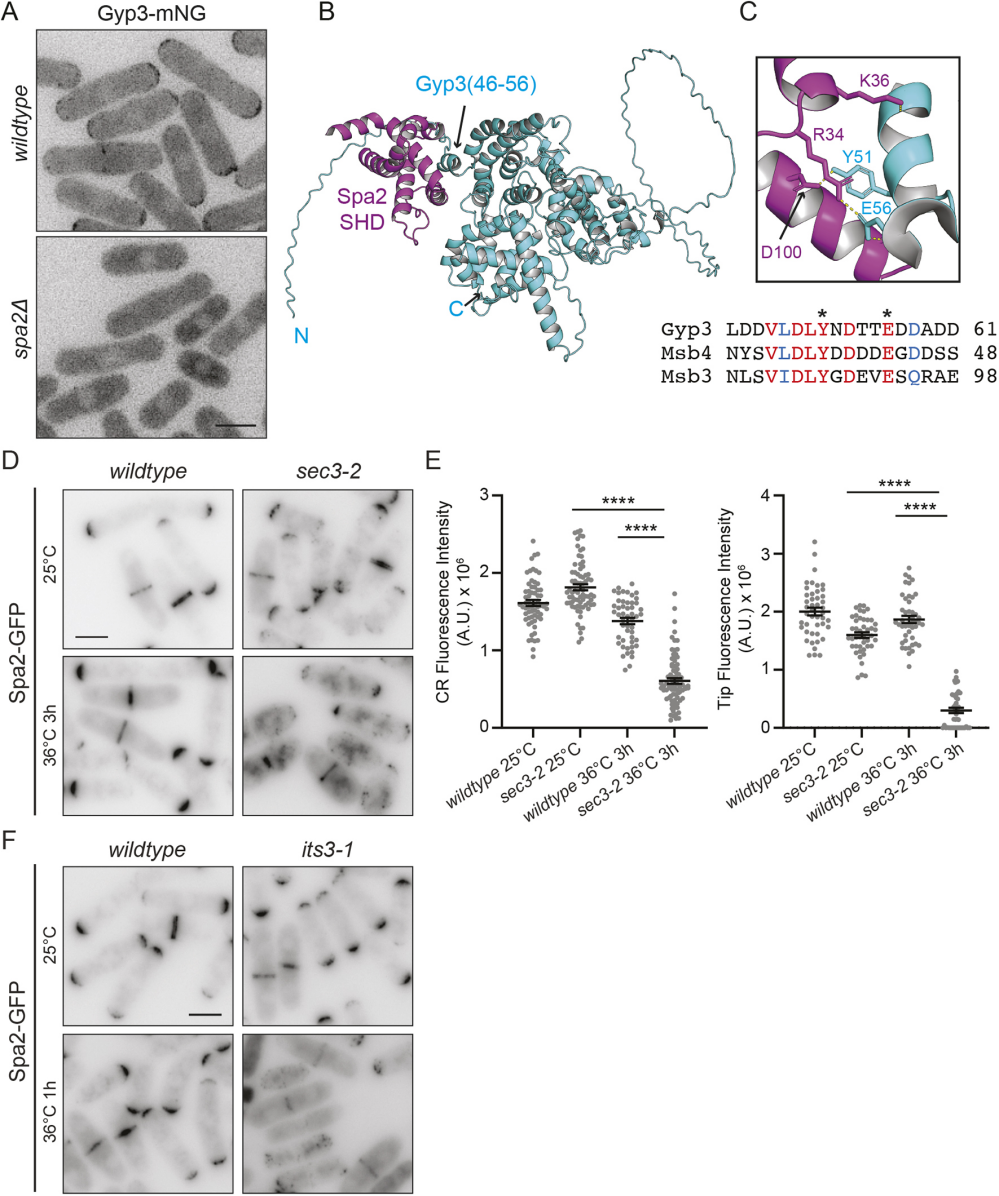
**Spa2 localization to cell tips and the cell division site depends on exocytosis.** (A) Representative live-cell images of Gyp3–mNG in wild-type and *spa2*Δ cells. Images are representative of three independent experiments. (B,C) AF3 model of Gyp3–Spa2 SHD interaction. In C, the predicted interaction region is enlarged and a sequence comparison of the interacting motif in the related Rab GAP proteins is shown below. Residues in red are identical and those in blue are similar. Asterisks denote the residues involved in direct interaction with the SHD domain. (D) Representative live-cell images of Spa2–GFP in wild-type and *sec3-2* cells. The strains were grown at 25°C and shifted to 36°C for 3 h and imaged at both temperatures. (E) Quantification of CR (left) or cell tip (right) fluorescence intensity of the strains in D. The graphs represent data from three separate experiments with *n*≥44 for each strain and position. Error bars represent mean±s.e.m. ****P<0.0001 (unpaired, two-tailed Student's *t*-test). A.U., arbitrary units. (F) Representative live-cell images of Spa2–GFP in wild-type and *its3-1* cells. The strains were grown at 25°C and shifted to 36°C for 1 h and imaged at both temperatures. Images are representative of three independent experiments. Scale bars: 5 µm.

The multimerization of Spa2–Pos1 is independent of the SHD and therefore cannot explain the necessity for the SHD in Spa2 localization. Thus, we examined whether analogous SHD domain-mediated interactions to those identified in other organisms influenced *S. pombe* Spa2 localization. In *S. cerevisiae,* the Spa2 SHD associates with components of a mitogen-activated protein kinase pathway and helps concentrate them at sites of active growth (Sheu et al., 1998; van Drogen and Peter, 2002). Spa2–GFP localized normally in the absence of *S. pombe* Mkh1 (Sengar et al., 1997), the most upstream kinase of the pathway and the only member of the pathway reported to localize to cell tips (Kanda et al., 2016) (Fig. S4A). Pek1 (also known as Skh1), the ortholog of *S. cerevisiae* Mkk1 and Mkk2, was not observed at tips, as previously reported (Madrid et al., 2006), and its localization was unaffected in *spa2*Δ cells (Fig. S4B). Although we did not detect mNeonGreen (mNG)-tagged Mkh1 (Mkh1–mNG) at cell tips, we did observe it at the septum, and this localization was unchanged in *spa2*Δ cells (Fig. S4B). Spa2 SHDs also bind Rab GAPs and are required for their polarized localization (Callejas-Negrete and Castro-Longoria, 2019; Lawson et al., 2022; Tcheperegine et al., 2005; Zheng et al., 2020). The single *S. pombe* cognate of these GAP proteins is Gyp3, a previously uncharacterized non-essential protein (Rutherford et al., 2024). Gyp3–mNG localization to cell tips and division sites was lost in *spa2*Δ cells (Fig. 5A), whereas Spa2–GFP tip and division site localization occurred normally in *gyp3*Δ cells (Fig. S4C). AF3 predicted a direct interaction between the Spa2 SHD domain and an α-helix near the N terminus of Gyp3 (Fig. 5B,C). The residues within Gyp3 (Y51 and E56) predicted to be key to this interaction are conserved within the fragments of *S. cerevisiae* Msb3 (amino acids 1–220) and Msb4 (amino acids 1–144) that bind directly to *S. cerevisiae* Spa2 (Tcheperegine et al., 2005) (Fig. 5C). Thus, we propose that the *S. pombe* Spa2 SHD domain similarly recruits Gyp3 to sites of polarized growth and division. While this paper was in revision, a similar conclusion regarding the importance of this short linear motif in interactions between Msb3/Msb4 and the Spa2 SHD was reached independently (Bareis et al., 2025 preprint). These results, however, do not explain how Spa2 itself is delivered to cell tips.

*S. cerevisiae* Spa2 also interacts with Bud6 and the formin Bni1, with which Bud6 associates to regulate F-actin cable formation; these interactions support budding yeast polarized localization (Fujiwara et al., 1998; Sheu et al., 1998). *S. pombe* Spa2–GFP localized to cell tips and the contractile ring (CR) in the absence of *S. pombe* Bud6 or the formin For3 (Fig. S4D,E), which is required for F-actin cable formation and myosin-mediated delivery of cargo to cell tips (Feierbach and Chang, 2001; Glynn et al., 2001). Reciprocally, Bud6 and For3 localized to cell tips and the CR at similar levels in *spa2*Δ cells as in wild-type cells (Fig. S4F,G). Indeed, *S. pombe* Spa2–GFP localization was largely independent of F-actin, as it persisted in cells treated with latrunculin A (LatA), a compound that causes F-actin depolymerization (Ayscough, 1998), whereas the localization of the F-actin patch protein Crn1 (Pelham and Chang, 2002) was dispersed by this treatment (Fig. S5A,B). Since the microtubule cytoskeleton is also involved in the proper morphogenesis of *S. pombe* cells (reviewed in Chiou et al., 2017), we analyzed Spa2 localization in cells treated with the microtubule-destabilizing drug, methyl benzimidazol-2-yl-carbamate (MBC). Although the cells became elongated and bent due to microtubule disruption (Hagan, 1998), Spa2 localized properly at cell tips and the division site (Fig. S5C). Like other tip-directed proteins, Spa2–GFP cortical localization was depolarized in mutants of the Orb6 kinase, which controls cell morphogenesis by spatially restricting Cdc42 to cell tips (Das et al., 2009), but it did not lose its cortical distribution (Fig. S5D).

Having ruled out cytoskeletal tracks as having a role in directing Spa2 to sites of cell growth, we tested whether Spa2 localization depends on the exocyst complex, because the *S. pombe* exocyst complex can localize to cell tips independently of cytoskeleton-based transport and its cortical localization is insensitive to LatA treatment (Bendezú and Martin, 2011; Nakano et al., 2011; Snaith et al., 2011). Sec3 and Exo70 are exocyst subunits that act in parallel to tether other exocyst components and secretory vesicles to the PM (Bendezú et al., 2012; He and Guo, 2009; Polgar and Fogelgren, 2018; Zuriegat et al., 2024). Spa2 localization to cell poles was normal in *exo70*Δ cells (Fig. S5E,F). However, at the restrictive temperature of *sec3-2* cells (Bendezú et al., 2012), Spa2–GFP was diminished at the cell division site by ∼56% and decreased at cell tips by ∼84% compared to levels in wild-type cells (Fig. 5D,E). Sec3 PM localization depends on its pleckstrin homology (PH) domain binding phosphatidylinositol-4,5-bisphosphate [PI(4,5)P_2_] (Bendezú et al., 2012). We reasoned that Spa2 localization should therefore also depend on PM PI(4,5)P_2_. To test this, we examined Spa2 localization in *its3-1* cells following just a 1 h shift to the non-permissive temperature. Its3 is the *S. pombe* phosphatidylinositol 4-phosphate 5-kinase that generates PM PI(4,5)P_2_ (Zhang et al., 2000). We found that Spa2 was lost from the cortex in *its3-1* cells (Fig. 5F). Taking these results together, we conclude that the exocyst complex plays a major role in targeting Spa2 to sites of polarized growth.

### Spa2 focuses the growth zone

**Fig. 6.**
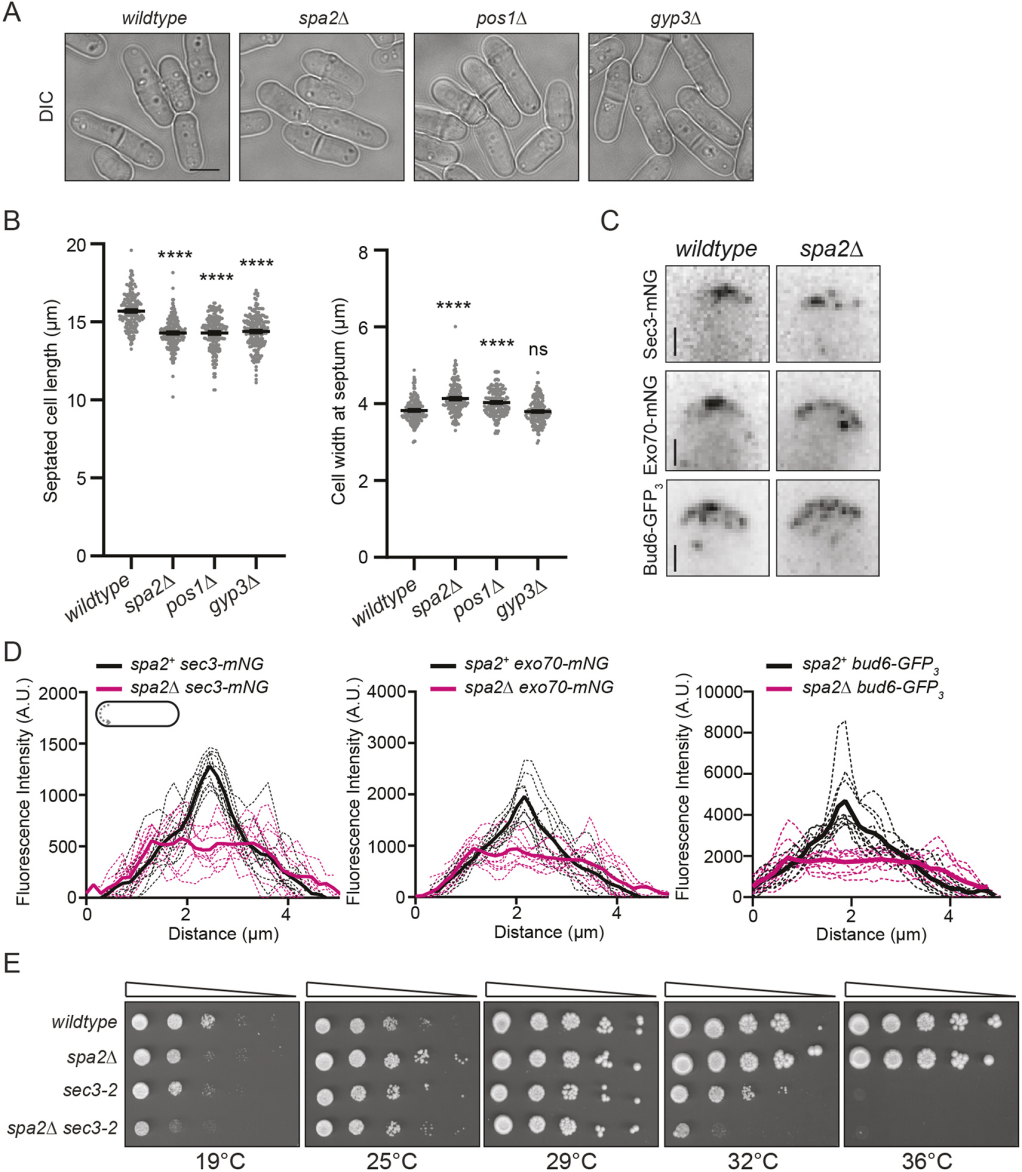
**Spa2 focuses the growth zone at cell tips.** (A) Live-cell DIC images of the indicated strains grown at 29°C in YE medium. Scale bar: 5 µm. (B) The length (left) and width (right) of cells from A at septation were measured. The graphs represent data from two independent experiments with *n*=200 for each strain and position. Error bars represent mean±s.e.m. ****P<0.0001; ns, not significant (unpaired, two-tailed Student's *t*-test compared to wild-type cells). (C) Representative live-cell images of Sec3–mNG, Exo70–mNG and Bud6–GFP_3_ in wild-type and *spa2*Δ cells. A zoomed in view of one cell tip from a bipolar cell is shown. Scale bars: 1 µm. (D) Fluorescence line scans drawn along the PM of the cell tip of bipolar cells for the indicated strains and indicated fluorescently tagged proteins. *n*=10 cells for each from two independent replicates. Solid lines represent the mean and dotted lines are the individual line traces. (E) The indicated strains were grown in liquid YE medium at 25°C until they reached mid-log phase and then adjusted to the same cell concentration measured by optical density. Next, 10-fold serial dilutions were made, and 2.5 µl of each was spotted on YE agar plates and incubated at the indicated temperatures for 2–3 days prior to imaging. Data shown are representative of two experiments.

By analogy with *S. cerevisiae*, Gyp3 acts on the Rab GTPase and Sec4 homolog Ypt2, which is required for exocytosis and therefore polarized growth (Craighead et al., 1993; Gao et al., 2003). Cdc42-GTP and Rho1-GTP might also be substrates (Tcheperegine et al., 2005). The fact that Gyp3 was completely dependent on Spa2 for its localization led us to consider that exocytosis, and therefore polarized growth, might be defective in *spa2*Δ cells. Upon careful examination, we found that *spa2*Δ cells and *pos1*Δ cells, which have less Spa2 at polarization sites, were reproducibly shorter and wider than wild-type cells and *spa2–GFP* cells (Fig. 6A,B; Fig. S5G). The *gyp3*Δ cells were also modestly shorter but not significantly wider (Fig. 6A,B). These observations suggest that particularly in the *spa2*Δ and *pos1*Δ strains, the growth zone is broader. To test this possibility, the localization of Exo70–mNG and Sec3–mNG was analyzed with line scans along the cell tips of bipolar *spa2*Δ cells. We found that not only were the Exo70–mNG and Sec3–mNG distributions broader in *spa2*Δ cells than in wild-type cells, but Exo70–mNG and Sec3–mNG lacked the medial peak of focused localization observed in wild-type cells (Fig. 6C,D). However, the overall levels of Exo70–mNG and Sec3–mNG at cell tips were unchanged in *spa2*Δ cells compared to wild-type cells (Fig. S5H,I). Bud6, the activator of formin For3, is transported to cell tips via the secretory machinery (Jin and Amberg, 2001). Therefore, we expected the tip distribution of Bud6–GFP_3_ to mirror that of Exo70–mNG and Sec3–mNG and to be broader in *spa2*Δ cells. This is what we observed (Fig. 6C,D), and there was no change in total Bud6–GFP_3_ cell tip localization in *spa2*Δ cells compared to that seen in wild-type cells (Fig. S4G). Consistent with a role in polarized exocytosis mediated by Sec3, *spa2*Δ showed a strong negative genetic interaction with *sec3-2* (Fig. 6E). Together, these results indicate that Spa2 is delivered to sites of cell growth primarily by the exocyst, and once there, it contributes to centering the zone of polarized exocytosis.

**Fig. 7.**
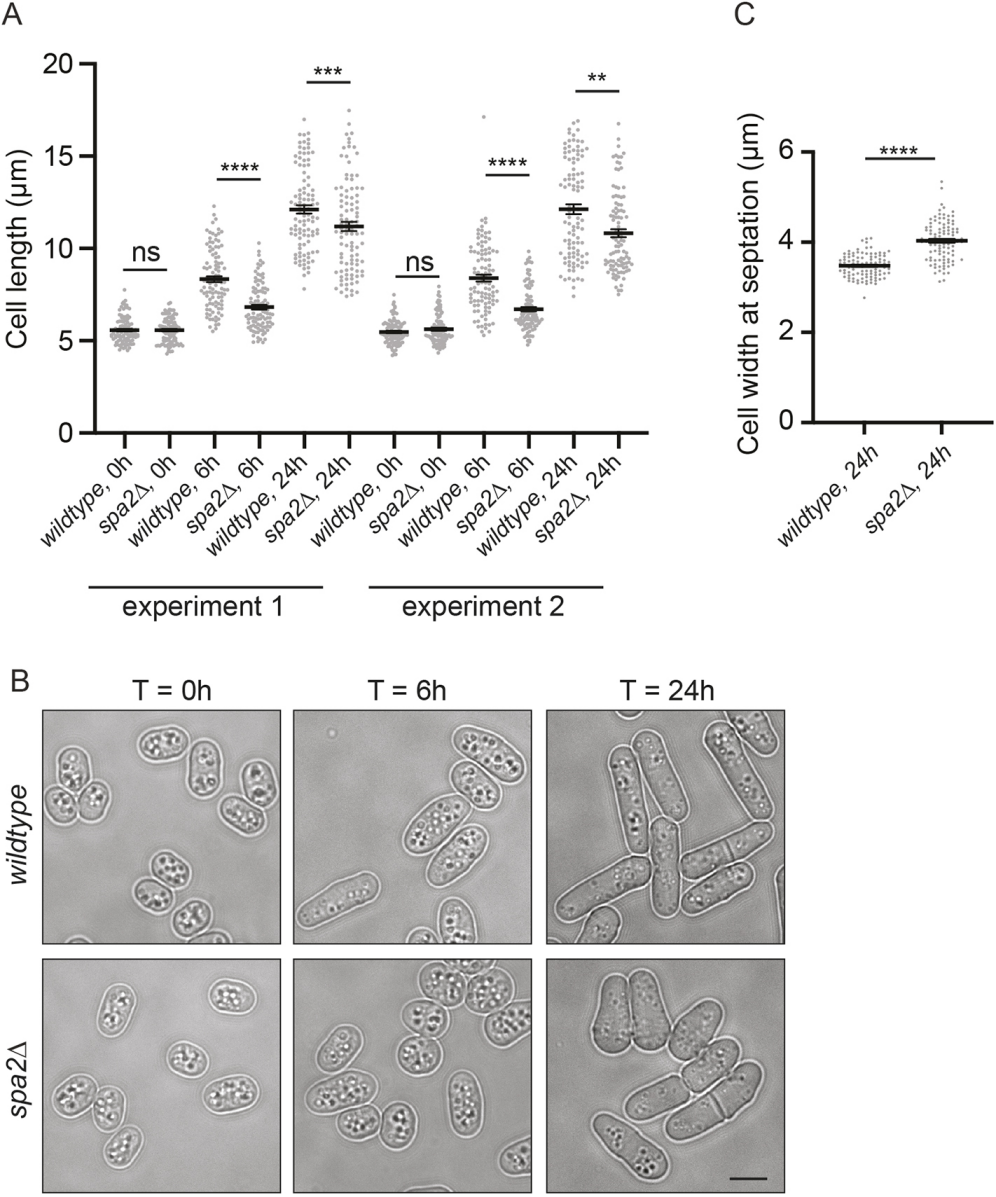
**Spa2 contributes to the resumption of cylindrical growth following starvation.** (A) Cell length measurements at 0 h, 6 h and 24 h after the resumption of growth at 32°C in EMM following 48 h nitrogen deprivation for wild-type and *spa2*Δ cells. The graph presents data from two independent experiments with *n*=100 for each strain and time point. Error bars represent mean±s.e.m. ****P<0.0001; ***P<0.001; **P<0.01; ns, not significant (unpaired, two-tailed Student's *t*-test). (B) Representative live DIC images of the indicated strains at the time points measured in A. Scale bar: 5 µm. (C) Width of wild-type and *spa2*Δ cells at septation following 24 h re-growth. The graph combines data from two independent experiments with *n*=100 for each strain and time point. Error bars represent mean±s.e.m. ****P<0.0001 (unpaired, two-tailed Student's *t*-test).

To determine whether the defects in steady-state polarized growth of *spa2*Δ cells at 32°C might be exacerbated when the polarity machinery is challenged, we starved wild-type and *spa2*Δ cells of nitrogen to induce cell rounding and growth arrest and then followed cell morphology upon re-feeding. After nitrogen starvation at 32°C for 48 h, wild-type and *spa2*Δ cells arrested growth at the same short size (Fig. 7A,B). Upon re-feeding at 32°C, we observed that wild-type cells began to adopt a typical cylindrical shape by 6 h. In contrast, *spa2*Δ cells maintained a shorter, rounded morphology for significantly longer before resuming cylindrical growth by 24 h (Fig. 7A,B). No septation was observed in either strain at the 6 h time point. The more rounded shape of *spa2*Δ cells translated to a significant difference in cell width in septating cells at 24 h (Fig. 7C). These results further establish a role for Spa2 in focusing the zone of polarized growth.

## DISCUSSION

Spa2 proteins in yeast and filamentous fungi are conserved cortical markers of cell polarization sites. Our comprehensive structure–function analysis of Spa2 localization in *S. pombe* reveals that it is governed by a modular set of conserved domains that act cooperatively and, in some cases, redundantly to tune Spa2 localization strength and distribution. Importantly, we identified the exocyst as essential for Spa2 positioning at growth sites and show that Spa2 contributes to focusing the growth zone, revealing a previously underappreciated but conserved role for this protein in sharpening the axis of vegetative growth*.* Together with our phylogenetic analysis of Spa2 protein organization and interactomes, our results indicate that the polarity networks scaffolded by Spa2 have been significantly rewired over the course of evolution.

Spa2 proteins have an overarching conserved architectural plan comprising the SHD, CC1, CC2 and FAT domains (Fig. S1A). Our domain-level dissection has defined how each of these regions affects the spatial distribution of *S. pombe* Spa2, adding to previous knowledge of the role played by a PXXP motif lying between domains that interacts with F-BAR proteins Cdc15 and Imp2 (Ren et al., 2015). As in other Spa2 proteins, the SHD is essential but not sufficient for polarized localization. We also found that the SHD is used to interface with Gyp3, the Rab GAP for the Sec4 homolog Ypt2; this interaction is conserved in *S. cerevisiae* and *N. crassa* (Callejas-Negrete and Castro-Longoria, 2019; Lawson et al., 2022; Tcheperegine et al., 2005; Zheng et al., 2020), and echoes the role of GIT proteins in recruiting GAPs in mammalian cells (Zhou et al., 2016). This spatial control of Rab activity would ensure that vesicle fusion (and delivery of cell wall enzymes) is concentrated where Spa2 resides, reinforcing a sharp boundary between growing and non-growing regions of the cell surface. While the hallmark SHD might have evolved to interface with small GTPase regulators across eukaryotes, neither SHD–GAP interactions nor other reported SHD-mediated protein interactions can fully explain the requirement for the SHD for Spa2 cortical localization (this study and Xie and Miao, 2021), leaving the major targeting role of the SHD in Spa2 proteins unresolved.

We have shown that the CC and FAT domains, although dispensable individually, work redundantly and amplify Spa2 enrichment at the cortex, in part by supporting oligomerization. Like its human homolog GIT1 (Paris et al., 2003; Premont et al., 2004; Zhu et al., 2020), Spa2 self-associates via its CC domains, and this dimerization is augmented through a bridging interaction with dimeric Pos1, forming a higher-order complex. Our results raise the possibility that Spa2–Pos1 multimers represent a rudimentary polarity condensate, effectively increasing the local concentration of Spa2 and its partners. Although we have shown that the FAT domains facilitate Spa2 localization in collaboration with CC-mediated oligomerization, more work needs to be done to understand the molecular basis of their conserved functions.

Our comparative analysis underscored the diversity of reported Spa2 binding partners across organisms (Fig. S1B). For example, *S. pombe* Spa2 associates with a distinct set of partners, notably the F-BAR protein Cdc15 and the fission yeast-specific Pos1 (Ren et al., 2015). Such non-uniform interactomes are difficult to reconcile with a general definition of a discrete and conserved ‘polarisome’ complex, as defined in budding yeast (Xie and Miao, 2021). Importantly, an equivalent of Pea2, which is considered central to the polarisome complex and links *S. cerevisiae* Spa2 to type V myosin (Dünkler et al., 2021), does not exist in *Aspergillus*, *Ustilago*, *Neurospora* or *Schizosaccharomyces* species (Fig. S1B). Thus, whether Spa2 proteins are physically connected to the actin cytoskeleton in organisms other than budding yeast is unclear. Indeed, in *S. pombe*, which lacks homologs of Pea2 and the actin nucleator Aip5 (Rutherford et al., 2024), we found that Spa2 localization does not rely on the actin cytoskeleton. Instead, the exocyst appears to be the principal determinant of Spa2 positioning. As the exocyst can itself localize to cell tips in the absence of cytoskeletal input in *S. pombe* (Bendezú and Martin, 2011), this pathway might represent an evolutionarily ancient mechanism for anchoring Spa2 complexes at the cortex. It also suggests a feed-forward loop in which the exocyst can transport and tether Spa2 to the PM, and Spa2 in turn recruits Gyp3 to restrict the spatial domain of exocytosis. This would be similar to the positive feedback loop involving Spa2 in *S. cerevisiae* (Lawson et al., 2022), although without the contribution of actin nucleators. Although such a model is consistent with our data, the mechanism by which Spa2–Gyp3 interaction influences exocyst positioning remains to be defined. It is also unclear whether the dependence of Spa2 on the exocyst reflects direct physical recruitment or a secondary consequence of impaired vesicle trafficking.

Despite differences in interactomes, a commonality among Spa2 proteins is their function in confining the zone of polarized growth (Carbó and Pérez-Martín, 2008; Knechtle et al., 2003; Sheu et al., 1998; Zheng et al., 2003), and this study positions *S. pombe* Spa2 function similarly. Spa2 might also perform this function during mating or when cells are under stress, but this role remains masked by redundant polarity pathways. This unifying view contextualizes long-standing observations of the variable importance of Spa2 proteins for morphogenesis across species and helps redefine the role of Spa2 from a component of an ill-defined polarisome complex to a broadly conserved spatial regulator of the growth machinery.

## MATERIALS AND METHODS

### Yeast strains and media

*S. pombe* strains (Table S1) were grown in yeast extract (YE) medium or minimal medium (EMM) with appropriate supplements (Moreno et al., 1991) and are available upon request. Transformations were performed by the lithium acetate method (Gietz et al., 1995; Keeney and Boeke, 1994). Epitope-tagged strains were constructed as described previously (Bähler et al., 1998; Wach et al., 1994) so that open reading frames were tagged at the 3′end of endogenous loci or at internal amino acids with the *GFP-kanMX6*, *mNG-kanMX6*, *mNG-hphMX6* or *FLAG-kanMX6* cassettes. G418 (Geneticin, 100 µg/ml; Thermo Fisher Scientific, 11811031) or hygromycin B (50 µg/ml; Life Technologies, 10687010) was used for selection of strains containing appropriate tags. Appropriate tagging was confirmed by PCR, live-cell imaging and/or immunoblotting. Strain construction and tetrad analyses were accomplished through standard methods (Forsburg and Rhind, 2006). Unless otherwise noted, cells used for live-cell imaging were grown at 25°C in YE medium. For yeast expression, fragments of *spa2* were inserted into pREP vectors containing the thiamine-repressible *nmt81* promoter (pREP81) (Basi et al., 1993). Transformants were grown in the presence of 5 µM thiamine to dampen expression and then washed into thiamine-free EMM to allow gene expression.

Directed yeast two-hybrid assays were done as described previously using *S, cerevisiae* strain PJ69-4A and the pGAD424 and pGBT9 plasmids with growth selection on media lacking histidine (James et al., 1996).

To depolymerize the actin cytoskeleton, a stock solution of latrunculin A (Focus Biomolecules) at 5 mM in DMSO was made and was added to 1 ml of a yeast culture for a final concentration of 100 μM. The cells were incubated for 10 min prior to imaging. To depolymerize the microtubules, a stock solution of 2.5 mg/ml of methyl benzimidazol-2yl carbamate (MBC; Sigma) was made in DMSO. The stock of MBC was added to 1 ml yeast culture at 1:100 dilution for a final concentration of 25 μg/ml. The cells were incubated for 15 min prior to imaging. The same volume of DMSO was added to 1 ml yeast cultures for controls.

For nitrogen starvation experiments, wild-type and *spa2*Δ cells were grown to mid-log phase, switched from EMM medium to EMM medium lacking nitrogen by filtration, and incubated with shaking at 32°C for 48 h. Cells were then diluted to an optical density at 600 nm (OD600) of 0.3 and kept in mid-log phase at 32°C by addition of new medium for 24 h. Samples were collected at 0 h, 6 h and 24 h for live-cell differential interference contrast (DIC) imaging. The length of cells was measured at each time point, and the width of cells that had reached septation at 6 h and 24 h was also measured. The experiment was performed twice.

The *spa2* open reading frame with 500 bp of the 5′ untranslated region (UTR) and 500 bp of the 3′ UTR was cloned into pIRT2 (Booher and Beach, 1986) using the BamHI and PstI restriction sites. Point mutations in *spa2* were made by PCR-mediated mutagenesis of pIRT2-*spa2* using *PfuTurbo* DNA polymerase (Agilent, 600252). Spa2 C-terminal truncations were generated by PCR-mediated insertion of GFP after the specified residues within the open reading frame. Spa2 internal deletions were made by synthesizing gene blocks (Integrated DNA Technologies) that contained the desired deletions, 300 bp of the 5′ UTR and 300 bp of the 3′ UTR. These DNA fragments were cloned into the PstI site of the pIRT2 plasmid using Gibson assembly. Successful cloning was confirmed by whole-plasmid sequencing. The gene deletions or point mutants were amplified from the relevant vector and transformed into the *spa2::ura4^+^* strain, and colonies were selected with 5-fluoroorotic acid (5-FOA, 1.5 mg/ml; US Biological). All *spa2* alleles were confirmed by whole-cell PCR and amplifying the gene from genomic DNA and sequencing. PCR products and plasmids were sequenced by Plasmidsaurus using Oxford Nanopore Technology with custom analysis and annotation.

### Microscopy

Live-cell images of *S. pombe* cells were acquired using (1) a personal DeltaVision microscope system (GE Healthcare, Issaquah, WA, USA), which includes an Olympus IX71 microscope, 60× NA 1.42 PlanApo and 100× NA 1.40 UPlanSApo objectives, fixed- and live-cell filter wheels, a Photometrics CoolSnap HQ2 camera and softWoRx imaging software or (2) a Zeiss Axio Observer inverted epifluorescence microscope with a Zeiss 63× oil (1.46 NA) objective, Zeiss ZEN 3.0 (Blue edition) software and an Axiocam 503 monochrome camera (Zeiss).

To directly compare wild-type and mutant populations within the same field of view, one population was incubated with fluorescently conjugated lectin (Sigma-Aldrich), which labels cell walls. Specifically, 1 μl of a 5 mg/ml stock of fluorescein isothiocyanate (FITC)–lectin in water was added to 1 ml of cells for a final concentration of 5 μg/ml. Cells were then incubated for 10 min at room temperature, washed three times in YE medium, and resuspended in YE medium. The lectin-labeled cell population and unlabeled cell population were mixed 1:1 immediately before imaging. The reciprocal labeling of populations was also done to account for any signal bleed through.

Intensity measurements were made with ImageJ software (https://imagej.net/software/fiji/) (Schindelin et al., 2012). For all intensity measurements, the background was subtracted by creating a region of interest (ROI) in the same image where there were no cells (Waters, 2009). Measurements of protein localization were done in two or three biological replicates and images were acquired using *Z*-series optical sections of 0.5 μm spacing for a total of 4.5–5.0 μm. Images for quantification were not deconvolved and sum projected. For cell tip intensity measurements, an ∼4.5 μm diameter circle ROI was applied to cell tips to acquire an intensity measurement. For line scans in Fig. 6, intensity measurements of ten cells with a line drawn along the cell tip were plotted against distance.

Cell lengths and widths were measured from single medial slices using Fiji (Schindelin et al., 2012). Line segments between each pole of the cell along the midpoint of the diameter were drawn to determine cell lengths at septation. Similarly, line segments across complete septa were drawn to determine cell widths.

FRAP analysis was performed on a Leica Thunder Imager system including a DMi8 inverted microscope, a 63× plan apo oil objective (1.40 NA), a Leica K8 sCMOS camera, standard excitation and emission filters, and an LED light source. Images were captured using Leica Application Suite X (LAS X) software. Spa2–GFP at a cell tip was partially or wholly photobleached with a 488 nm laser. Images were acquired every 1 s for 30 s, and three images were taken prior to the photobleaching event. Quantifications of the fluorescence recovery were performed using ImageJ. FRAP intensity measurements were corrected for background and time course photobleaching. Each FRAP measurement was obtained from a separate cell, and 14 cells were analyzed in three separate experiments.

### Immunoprecipitations and immunoblotting

Whole-cell lysates were prepared in NP40 buffer in native conditions as previously described (Gould et al., 1991). Proteins were immunoprecipitated from protein lysates using 4 µg/ml anti-GFP (Roche, Nutley, NJ, USA) or 2 µg/ml anti-FLAG (Sigma) followed by Protein G Sepharose beads (GE Healthcare).

For immunoblotting, proteins were resolved by 10% PAGE or 4–12% NuPAGE, transferred by electroblotting to a polyvinylidene difluoride membrane (Immobilon P; Millipore Corp., Bedford, MA, USA) and incubated with the set of primary antibodies indicated at 1 µg/ml. Primary antibodies were detected with secondary antibodies coupled to Alexa Fluor 680 (Life Technologies, Grand Island, NY, USA) or IRDye800 (LI-COR Biosciences, Lincoln, NE, USA) and visualized using an Odyssey Infrared Imaging System (LI-COR Biosciences).

### AlphaFold3 structural prediction

Protein structure predictions were generated with the AlphaFold3 server (https://alphafoldserver.com; Abramson et al., 2024). Protein sequences from PomBase (Rutherford et al., 2024) were entered into the server with the indicated copies of each protein. The automatic seed setting was used for all predictions. For protein–protein interaction analysis, the top-ranked ‘model_0’ prediction was selected to extract interface information and visualization using the PyMOL molecular graphics system (version 3.0, Schrodinger, LLC).

### Statistical analysis

All statistical analyses were performed in Prism 8 (Graphpad software). No data were excluded from the analysis.

### Recombinant protein production and purification

Spa2(305–360) and Pos1 cDNA fragments were amplified from plasmids and inserted into pMAL-c2 (New England Biolabs) and pGEX4T-1 (Cytiva), respectively, using the Gibson assembly technique (Gibson et al., 2009). Spa2(200–360) and Pos1 constructs were also cloned into pETDuet (Millipore Sigma) using the Gibson assembly technique (Gibson et al., 2009). Plasmids were transformed into DH5α *Escherichia coli*, and all constructs were verified by sequencing. MBP–Pos1, GST–Spa2(305–360), FLAG–Spa2(200–360) and/or His_6_–Pos1 proteins were produced in Rosetta2(DE3)pLysS bacteria (Novagen) grown in Terrific broth (23.6 g/l yeast extract, 11.8 g/l tryptone, 9.4 g/l K_2_HPO_4_, 2.2 g/l KH_2_PO_4_, 4 ml/l glycerol) supplemented with 100 μg/ml ampicillin and 34 μg/ml chloramphenicol by incubating on ice for 15 min, adding 0.4 mM isopropyl β-D-1-thiogalactopyranoside (IPTG) (Fisher Scientific; BP1755), and incubating the cells for 16–18 h at 18°C. Cells were lysed by sonication.

To purify MBP and GST fusion proteins, frozen cell pellets were lysed in either MBP buffer [20 mM Tris-HCl pH 7.4, 150 mM NaCl, 1 mM EDTA, protease inhibitor tablets (Roche)] or GST buffer (4.3 mM NaHPO_4_, 137 mM NaCl, 2.7 mM KCl, 1 mM dithiothreitol) with the addition of cOmplete EDTA-free protease inhibitor cocktail (Roche; 05056489001) and 0.1% NP-40 (US Biologicals; N3500). GST–Spa2(305–360) was purified with GST-bind resin (Millipore, 70541) in the same buffer. MBP–Pos1 was purified on amylose beads (New England Biolabs) in column buffer [20 mM Tris-HCl pH 7.4, 150 mM NaCl, 1 mM EDTA, protease inhibitor tablets (Roche)] and eluted in the same buffer with 10 mM maltose. For the binding reaction, GST proteins were left on beads and incubated with eluted MPB proteins in GST buffer for 40 min at 4°C. Beads were washed in GST buffer three times and then SDS-PAGE sample buffer (0.25 M Tris-HCl, pH 8.8, 20% glycerol, 4% SDS, 20% β-mercaptoethanol, 0.5 mg/ml Bromphenol Blue) was added. The mixture was heated to 95°C, separated on a NuPAGE 4–12% Nu-PAGE gel (MOPS buffer), stained with Coomassie Blue, and imaged with an Odyssey CLx instrument (LI-COR Biosciences).

FLAG–Spa2(200–360) alone or FLAG–Spa2(200–360) with His_6_–Pos1 was purified on ChromoTek DYKDDDDK Fab-Trap Agarose (Proteintech, ffa) in 10 mM Tris-HCl pH 7.4, 150 mM NaCl, 0.5 mM EDTA, 0.5% NP-40, 1 mM PMSF and protease inhibitor cocktail (Roche), and washed three times in the same buffer except a lower concentration of NP-40 (0.05%). Proteins were eluted with the addition of 150 µg/ml of 3×FLAG peptide in 10 mM Tris-HCl pH 7.5, 150 mM NaCl, 0.5 mM EDTA.

His_6_–Pos1 alone was purified on PureProteome nickel magnetic beads (EMD Millipore, LSKMAGH10) in 50 mM sodium phosphate, pH 7.8, 300 mM NaCl, 5 mM imidazole, 0.1% NP-40, 1 mM PMSF and protease inhibitor cocktail (Roche). His_6_–Pos1 was eluted in the same buffer except that the imidazole concentration was increased to 300 mM.

### Gel filtration chromatography

To analyze the size of protein complexes, gel filtration chromatography was used. A 500 µl volume containing 75 µg His_6_–Pos1, 100 µg FLAG–Spa2(200–360) or 111–188 µg of each protein together was applied to a Superose 6 10/300 GL high performance column of an AKTA pure system (Cytiva). Each protein complex was fractionated alone and again with two size calibration standards included (GEHealthcare LMW 28-4038-41 and HMW 28-4038-42). For each run at a flow rate of 0.5 ml/min at 4°C, 60 fractions of 500 µl were collected using elution buffer (10 mM Tris-HCl pH 7.5, 150 mM NaCl, 0.5 mM EDTA). Eluted fractions were analyzed by absorbance at 280 nm and by SDS-PAGE and Coomassie Blue staining. Molecular masses of the protein complexes were estimated from the standard curve of the calibration standards. These included thyroglobulin at 669 kDa, ferritin at 440 kDa, aldolase at 158 kDa, conalbumin at 75 kDa, ovalbumin at 44 kDa and carbonic anhydrase at 29 kDa.

### Artificial intelligence

ChatGPT-4o (OpenAI) was used to review the literature for any missing Spa2 references, point out potential errors or incompleteness in referencing within the text, propose titles, propose abstracts of various lengths, and to propose discussion topics and text based on the completed manuscript. All suggestions were critically reviewed and some proposed suggestions were found to be correct and useful. The authors take full responsibility for the content of this article.

## Supplementary Material

10.1242/joces.264071_sup1 Supplementary information
