# Supplementary material for: Oligomerization and exocyst coupling underlie Spa2-mediated focusing of polarized growth in fission yeast
Source: J Cell Sci. 2025 Sep 11;138(17):jcs264071. doi: 10.1242/jcs.264071 (PMC12450465; doi:10.1242/jcs.264071)
Supplement: Supplementary information [file joces-138-264071-s1.pdf]

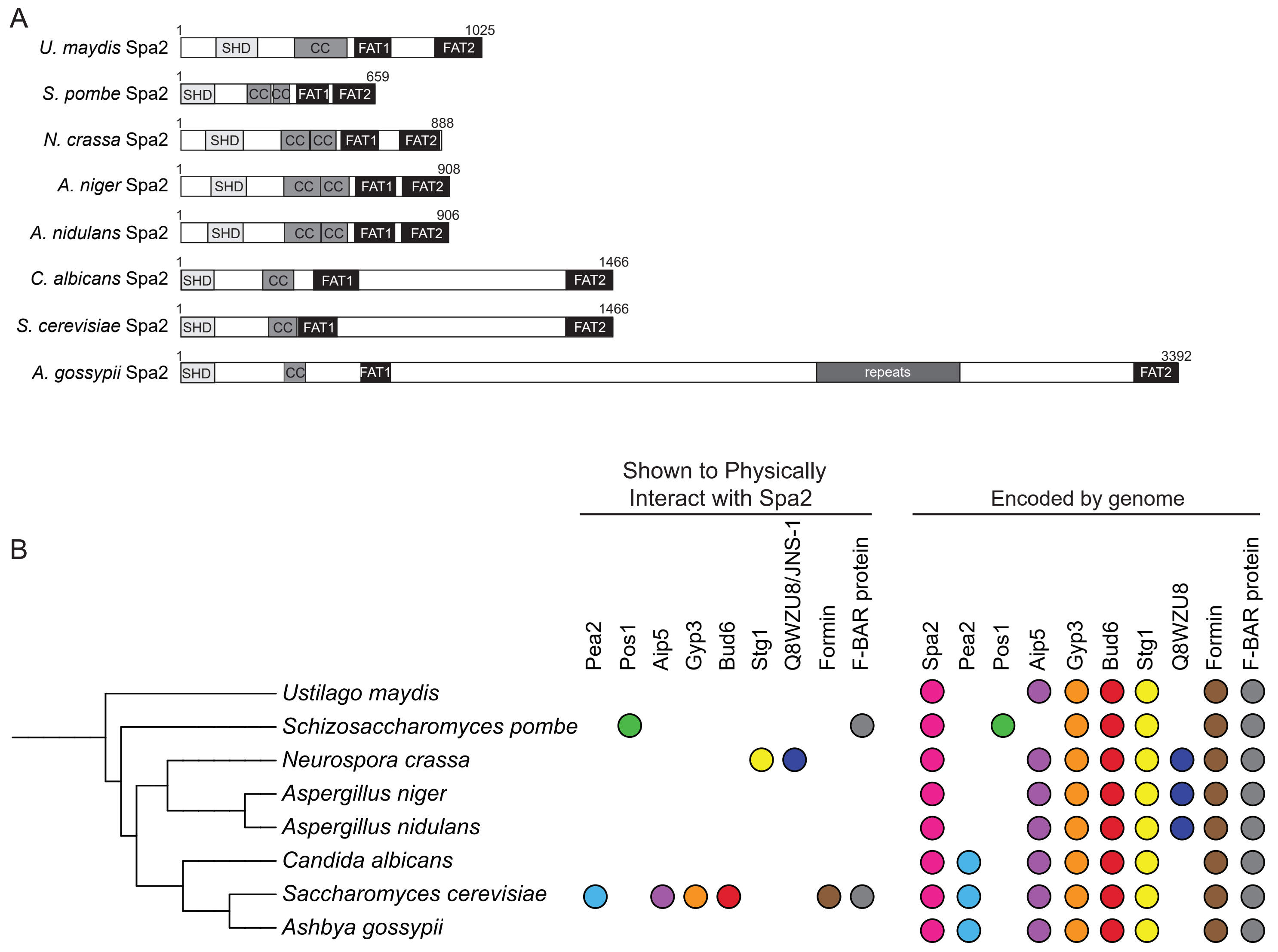

Fig. S1. Comparison of Spa2 and its interacting proteins among several fungal species. A) Schematic diagrams of the indicated most-studied Spa2 proteins drawn to scale with the Spa2 homology domain (SHD), coiled-coil (CC) domains, and focal adhesion targeting (FAT) domains indicated. A repeat region in *A. gypsii* is also indicated. B) Phylogenetic tree of select organisms in which Spa2 proteins have been studied (left) with the selected proteins that have been biochemically or by two-hybrid analysis identified as physically interacting with Spa2 in at least one organism (middle): Pea2 (Sheu et al., 1998); Pos1 (Ren et al., 2015); Aip5 (Glomb et al., 2019; Shih et al., 2005; Xie et al., 2019); Gyp3 (Tcheperegine et al., 2005); Bud6 (Sheu et al., 1998); Stg1 (Zheng et al., 2020); Q8WZU8/JNS-1 (Zheng et al., 2020); formin (Fujiwara et al., 1998); F-BAR proteins (Foltman et al., 2018; Moreno et al., 2013; Ren et al., 2015). (Right): the existence of the protein encoded by the genomes of the selected organisms. Each colored circle denotes a protein or putative homolog identified by protein BLAST search (Altschul et al., 1990; Engel et al., 2024).

Journal of Cell Science • Supplementary information

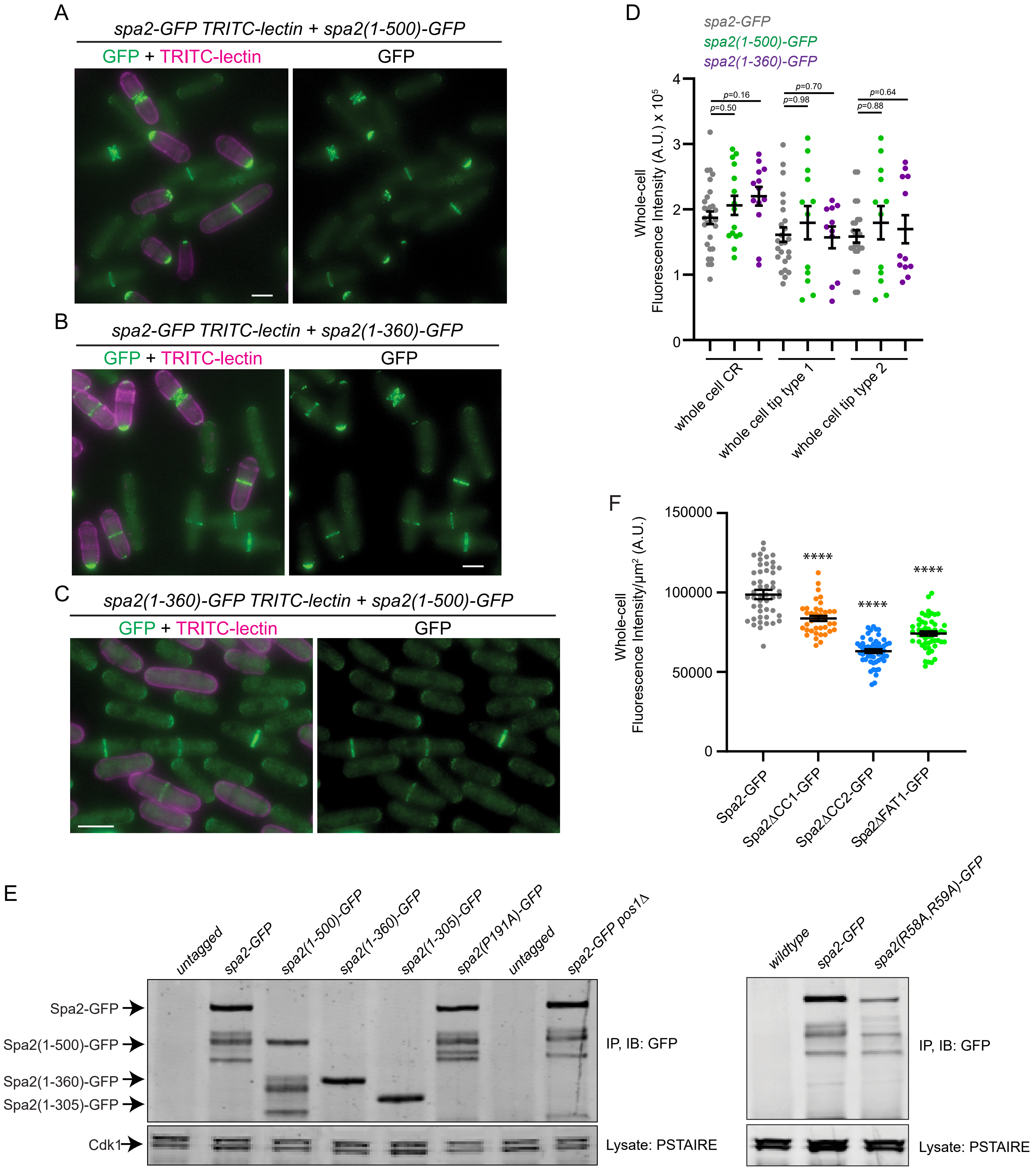

Journal of Cell Science • Supplementary information

Fig. S2. Comparison of the localization and levels of Spa2 mutants. A-B) *spa2-GFP* cells were labeled with TRITC-lectin and mixed with either *spa2(1-500)-GFP* cells (A) or *spa2(1-360)-GFP* cells (B) and imaged live. C) *spa2(1-360)-GFP* cells were labeled with TRITC-lectin and mixed with *spa2(1-500)-GFP* cells and imaged live. D) Quantitation of fluorescence intensity of whole cells with Spa2 at CRs, one tip (cell type 1) or two tips (cell type 2). Data is from two separate experiments and $n \geq 11$ for each cell type (one-way ANOVA). ****$P<0.0001$. Error bars represent mean ± SEM. E) A portion of lysates made from the indicated strains were either subjected to immunoprecipitation (IP) followed by immunoblotting (WB) with anti-GFP antibodies (upper panels) or immunoblotted directly with anti-PSTAIRE as a loading control (lower panels). Immunoblots comparing the level of Spa2-R58A,R59A-GFP to that of Spa2-GFP were performed four times for quantification purposes. F) Quantification of whole cells with Spa2 at CRs from the indicated strains. Data is from two separate experiments and $n \geq 11$ for each strain. ****$P<0.0001$. Error bars represent mean ± SEM. Scale bars, 5 µm.

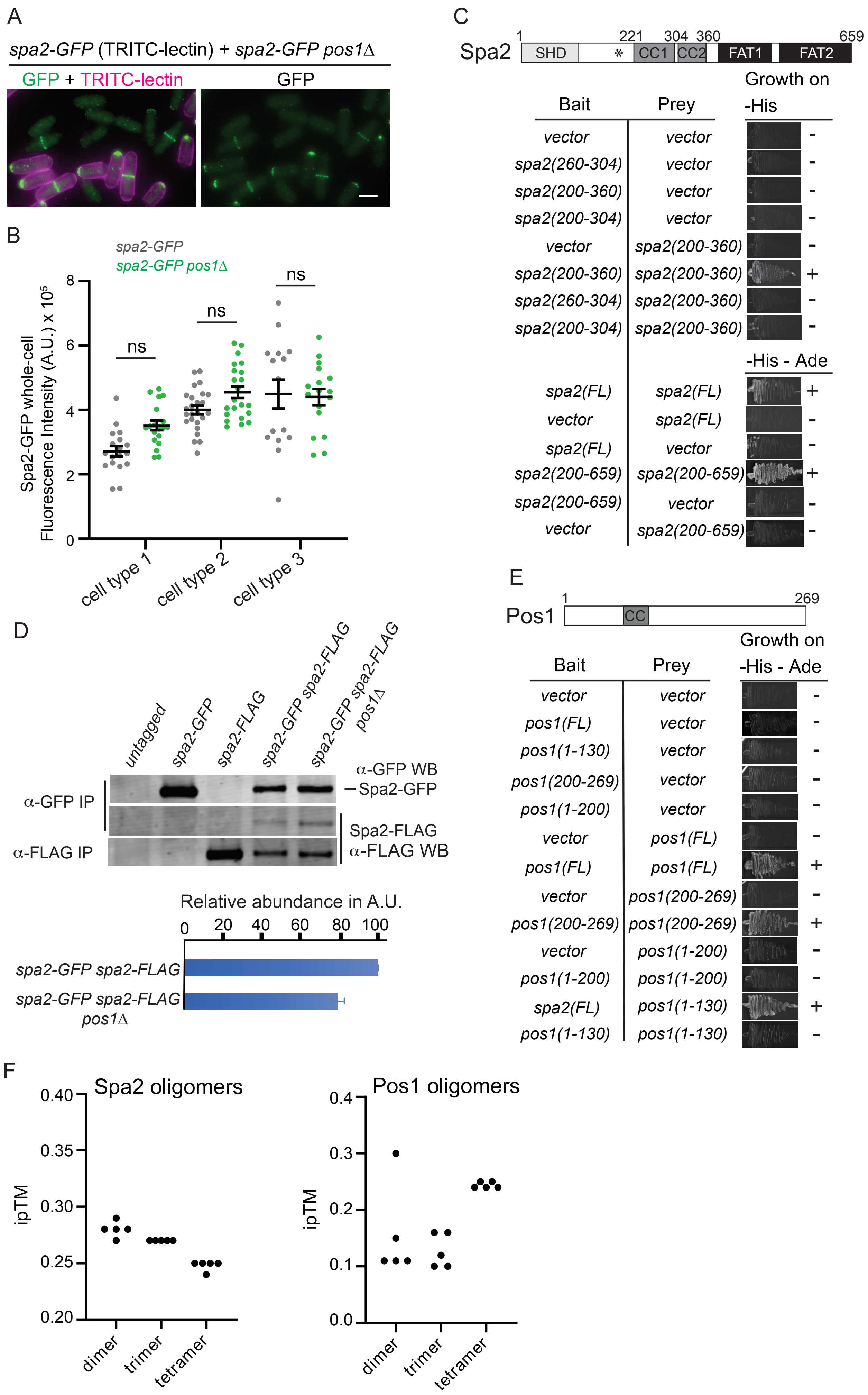

Journal of Cell Science • Supplementary information

Fig. S3. Characterization of Spa2 self- and Pos1-interactions. A) Representative live cell images of *spa2-GFP* cells labeled with TRITC-lectin and mixed with *spa2-GFP pos1Δ* cells. Scale bar, 5 µm. B) Quantitation of fluorescence intensity of whole cells with Spa2 at one cell tip (cell type 1), two cell tips (cell type 2), or the CR (cell type 3). Data is from two separate experiments and $n \geq 28$ for each cell type. Error bars represent mean ± SEM, (one-way ANOVA). C) Yeast two-hybrid assay results testing for spa2 self-interaction. Growth on media lacking adenine and histidine is shown and indicated with (+) for growth and (-) for no growth. D) An anti-GFP immunoblot (top panel) and an anti-FLAG immunoblot (middle and bottom panels) of anti-FLAG (bottom panel) or anti-GFP (top and middle panels) immunoprecipitations from the indicated strains. Spa2 self-interaction in the presence and absence of Pos1 was quantified using an Odyssey CLx instrument, normalized for background, and the levels in *pos1*Δ cells are presented relative to wildtype (histogram below the blots). E) Yeast two-hybrid assay results testing for *pos1* self-interaction and interaction with *spa2*. Growth on media lacking adenine and histidine is shown and indicated with (+) for growth and (-) for no growth. The majority of the yeast two-hybrid results presented in C, E and Fig. 3C are derived from experiments that were performed at the same time, and some streaks of relevant negative controls have been duplicated in these panels. F) Plots showing the individual ipTM scores for different oligomeric states (2-4) for Spa2 (left) and Pos1 (right). The predicted structures were generated with AlphaFold3 (Abramson et al., 2024).

Journal of Cell Science • Supplementary information

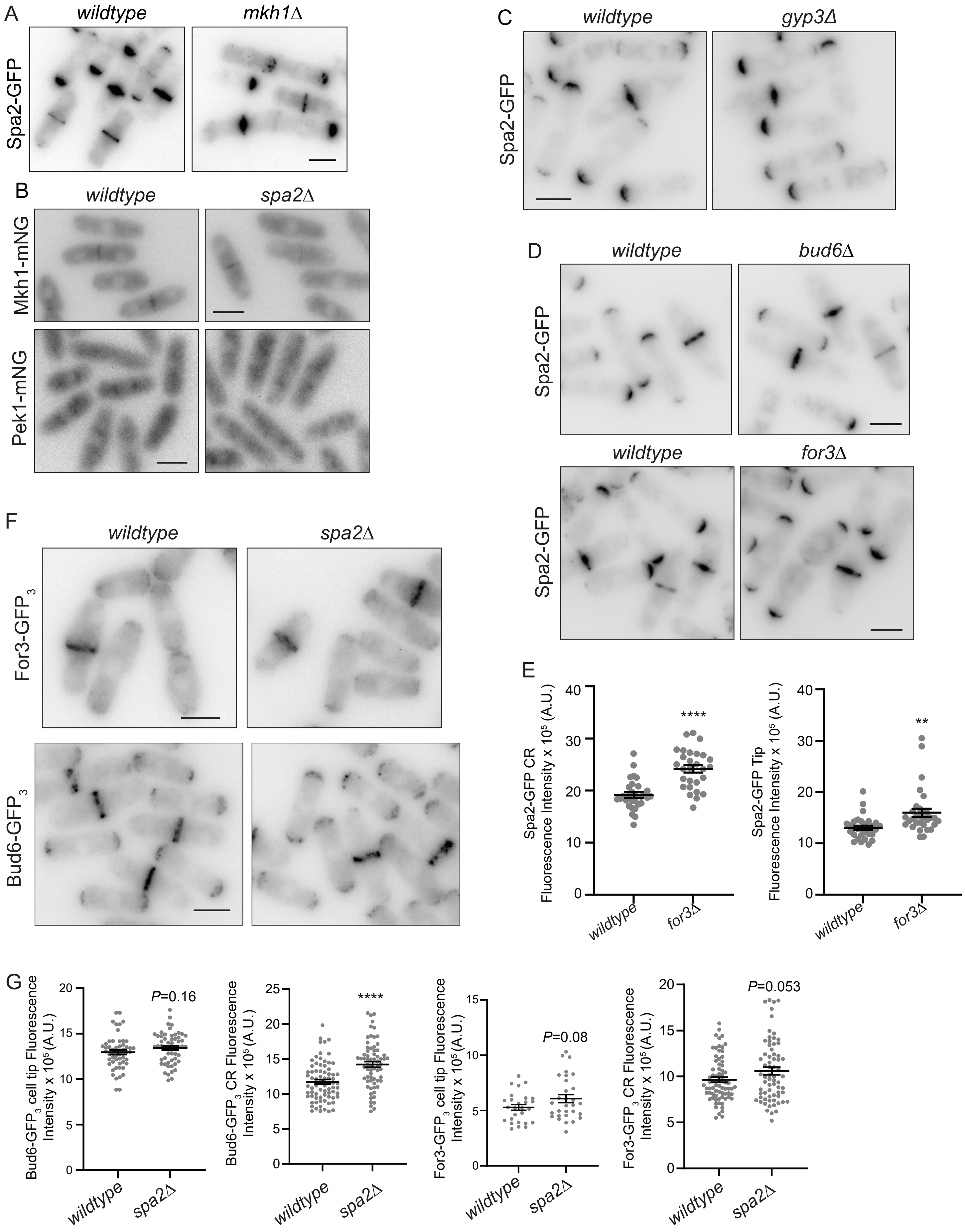

Fig. S4. Dependencies of Spa2 localization. A-D, F) Representative live cell images of the indicated proteins in the indicated strains. E) Quantification of CR or cell tip fluorescence intensity of Spa2-GFP in wildtype and *for3Δ* cells. The graphs represent data from two separate experiments and $n \geq 37$ for each strain. G) Quantification of CR or cell tip fluorescence intensity of Bud6-GFP$_3$ and For3-GFP$_3$ in wildtype or *spa2Δ* cells. The graphs represent data from two separate experiments and $n \geq 27$ for each strain. ****$P<0.0001$, **$P<0.01$ (unpaired, two-tailed Student's *t*-test). Error bars represent mean ± SEM. Scale bars, 5 μm.

Journal of Cell Science • Supplementary information

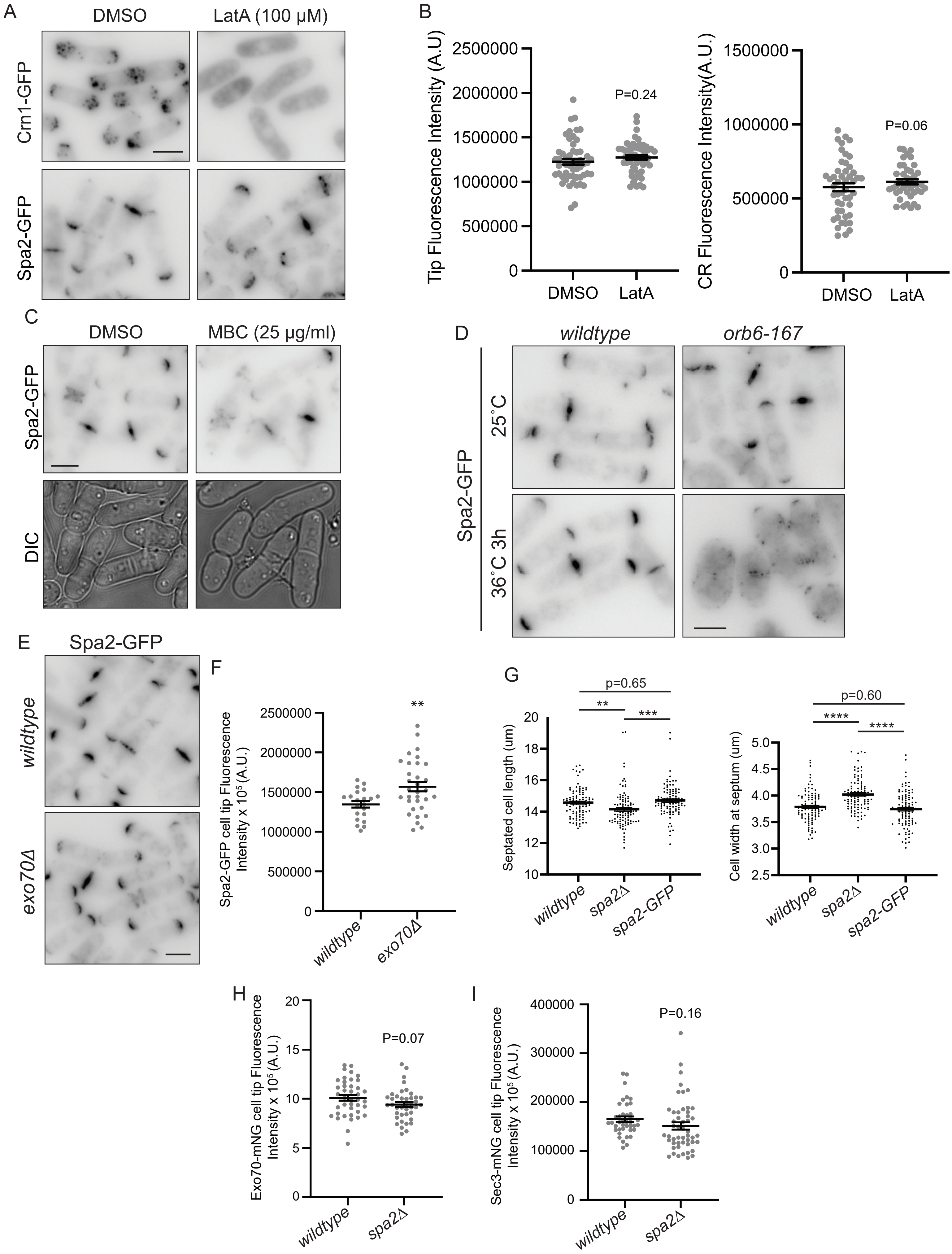
A
DMSO
LatA (100 μM)
Crn1-GFP
Spa2-GFP
B
Tip Fluorescence Intensity (A.U)
2500000
2000000
1500000
1000000
500000
0
P=0.24
DMSO
LatA
CR Fluorescence Intensity(A.U.)
1500000
1000000
500000
0
P=0.06
DMSO
LatA
C
DMSO
MBC (25 μg/ml)
Spa2-GFP
DIC
D
wildtype
orb6-167
Spa2-GFP
25˚C
36˚C 3h
E
Spa2-GFP
wildtype
exo70Δ
F
Spa2-GFP cell tip Fluorescence Intensity x 10⁵ (A.U.)
2500000
2000000
1500000
1000000
500000
0
**
wildtype
exo70Δ
G
Septated cell length (um)
20
18
16
14
12
10
p=0.65
**
***
wildtype
spa2Δ
spa2-GFP
Cell width at septum (um)
5.0
4.5
4.0
3.5
3.0
2.5
p=0.60
****
****
wildtype
spa2Δ
spa2-GFP
H
Exo70-mNG cell tip Fluorescence Intensity x 10⁵ (A.U.)
20
15
10
5
0
P=0.07
wildtype
spa2Δ
I
Sec3-mNG cell tip Fluorescence Intensity x 10⁵ (A.U.)
400000
300000
200000
100000
0
P=0.16
wildtype
spa2Δ

Fig. S5. Additional dependencies of Spa2 localization. A) Representative live cell images of Spa2-GFP and Crn1-GFP cells treated with DMSO or 100 µM LatA for 10 minutes. B) Quantification of CR or cell tip fluorescence intensity from bipolar cells of Spa2-GFP cells treated with DMSO or LatA as in A. The graphs represent data from three separate experiments and $n \geq 45$ for each strain (unpaired, two-tailed Student's *t*-test). Error bars represent mean ± SEM. C) Representative live cell images of Spa2-GFP cells treated with DMSO or 25 µg/ml MBC for 10 minutes. D) Representative live cell images of Spa2-GFP in *orb6-25* or *orb6-167*. Cells were grown at 25°C and then shifted to 36°C for 3 hours. Cells were imaged at both time points. Scale bars, 5 µm. E) Representative live cell images of Spa2-GFP in wildtype or *exo70Δ* cells. F) Quantification of cell tip fluorescence intensity of Spa2-GFP in wildtype or *exo70Δ* cells. The graphs represent data from two separate experiments and $n=40$ for each strain (unpaired, two-tailed Student's *t*-test). G) The lengths (left) and width (right) of the indicated strains at septation were measured. The graphs represent data from two independent experiments and $n=100$ for each strain and position. Error bars represent mean ± SEM. ****$P<0.0001$, ***$P<0.001$, **$P<0.01$ (one-way ANOVA). H-I) Quantification of cell tip fluorescence intensity of Exo70-mNG or Sec3-mNG in wildtype and *spa2Δ* cells. The graphs represent data from two separate experiments and $n \geq 40$ for each strain (unpaired, two-tailed Student's *t*-test).

Journal of Cell Science • Supplementary information

TABLE S1. ***S. pombe*** **strains used in this study**

**Figure 1**		
KGY15148	*spa2(1-500)-GFP:kanMX6 ade6-M21X ura4-D18 leu1-32 h$^-$*	This study
KGY15201	*spa2(1-360)-GFP:kanMX6 ade6-M21X ura4-D18 leu1-32 h$^-$*	This study
KGY15613	*spa2(1-305)-GFP:kanMX6 ade6-M21X ura4-D18 leu1-32 h$^-$*	This study
KGY15975	*spa2-GFP:kanMX6 ade6-M216 ura4-D18 leu1-32 h$^+$*	Lab stock
**Figure 2**		
KGY9250-2	*spa2Δ(CC1,221-301)-GFP:kanMX6 ade6-M21X ura4-D18 leu1-32 h$^+$*	This study
KGY9260-2	*spa2Δ(CC2,310-365)-GFP:kanMX6 ade6-M21X ura4-D18 leu1-32 h$^+$*	This study
KGY9374-2	*spa2Δ(FAT1,391-497)-GFP:kanMX6 ade6-M21X ura4-D18 leu1-32 h$^+$*	This study
KGY15819	*spa2-R58A,R59A-GFP:kanMX6 ade6-M21X ura4-D18 leu1-32 h$^-$*	This study
KGY15975	*spa2-GFP:kanMX6 ade6-M216 ura4-D18 leu1-32 h$^+$*	Lab stock
KGY16002	*spa2Δ::ura4$^+$ ura4-D18 leu1-32 ade6-210 h$^-$*	Lab stock
**Figure 3**		
KGY1296	*PJ69-4A MATa trp1-190 leu2-3,112 ura3-52 his3-200 gal4 delete gal80 delete LYS2 replaced by GAL1-HIS3 GAL2-ADE2 met2 replaced by GAL-lacZ*	Lab stock
KGY15975	*spa2-GFP:kanMX6 ade6-M216 ura4-D18 leu1-32 h$^+$*	Lab stock
KGY8379	*pos1-FLAG$_3$:kanMX6 ade6-M210 leu1-32 ura4-D18 h$^-$*	Lab stock
KGY9016	*spa2-GFP:kanMX6 pos1-FLAG$_3$:kanMX6 ade6-M210 leu1-32 ura4-D18 h$^-$*	This study
KGY9046	*spa2ΔCC2ΔFAT2-GFP:kanMX6 ade6-M21X ura4-D18 leu1-32 h$^-$*	This study
KGY11996	*spa2-GFP:kanMX6 pos1Δ::ura4$^+$ ade6-M21X ura4-D18 leu1-32 h$^-$*	This study
KGY15148	*spa2(1-500)-GFP:kanMX6 ade6-M21X ura4-D18 leu1-32 h$^-$*	This study
KGY15201	*spa2(1-360)-GFP:kanMX6 ade6-M21X ura4-D18 leu1-32 h$^-$*	This study
KGY15613	*spa2(1-305)-GFP:kanMX6 ade6-M21X ura4-D18 leu1-32 h$^-$*	This study
KGY15614	*spa2(1-360)-GFP:kanMX6 pos1-FLAG$_3$:kanMX6 ade6-M21X ura4-D18 leu1-32 h$^-$*	This study
KGY17284	*spa2(1-305)-GFP:kanMX6 pos1-FLAG$_3$:kanMX6 ade6-M21X ura4-D18 leu1-32 h$^-$*	This study
KGY17226	*spa2(1-500)-GFP:kanMX6 pos1Δ::ura4$^+$ ade6-M21X ura4-D18 leu1-32 h$^+$*	This study
**Figure 4**		
KGY7420	*spa2-GFP:kanMX6 ade6-M21X ura4-D18 leu1-32 h$^+$*	Lab stock
**Figure 5**		
KGY15975	*spa2-GFP:kanMX6 ade6-M216 ura4-D18 leu1-32 h$^+$*	Lab stock
KGY9292	*spa2-GFP:kanMX6 its3-1 ade6? leu1? ura4? h$^?$*	This study
KGY9388	*gyp3-mNG:kanMX6 ura4-D18 leu1-32 ade6-210 h$^-$*	This study

Journal of Cell Science • Supplementary information

KGY9389	gyp3-mNG:kanMX6 spa2Δ::ura4^{+} ura4-D18 leu1-32 ade6-210 h^{-}	This study
KGY9311-2	spa2-GFP:kanMX6 sec3-2:his5^{+}:ura4^{+} ade6-? leu1-32 ura4-D18 h^{-}	This study
**Figure 6**		
KGY246	ade6-M210 ura4-D18 leu1-32 h^{-}	Lab stock
KGY8906	bud6-GFP$_3$:kanMX6 ade6-M210 ura4-D18 leu1-32 h^{+}	(Glynn et al., 2001)
KGY9308-2	spa2Δ::ura4^{+} sec3-2:his5^{+}:ura4^{+} ade6-? leu1-32 ura4-D18 h^{-}	This study
KGY9375	gyp3Δ:kanMX6 ade6-M210 ura4-D18 leu1-32 h^{+}	Bioneer
KGY9395	spa2Δ::ura4^{+} bud6-GFP$_3$:kanMX6 ura4-D18 leu1-32 ade6-210 h^{-}	This study
KGY11951	pos1Δ::ura4^{+} ura4-D18 leu1-32 ade6-210 h^{-}	Lab stock
KGY1312-2	exo70-mNG:hphMX6 spa2Δ::ura4^{+} ura4-D18 leu1-32 ade6-210 h^{-}	This study
KGY1326-2	exo70-mNG:hphMX6 ura4-D18 leu1-32 ade6-210 h^{-}	This study
KGY1365-2	sec3-mNG:hphMX6 spa2Δ::ura4^{+} ura4-D18 leu1-32 ade6-210 h^{-}	This study
KGY1487-2	sec3-mNG:hphMX6 ura4-D18 leu1-32 ade6-210 h^{-}	This study
KGY16002	spa2Δ::ura4^{+} ura4-D18 leu1-32 ade6-210 h^{-}	Lab stock
YSM2070	sec3-2:his5^{+}:ura4^{+} ade6-? leu1-32 ura4-D18 h^{-}	(Bendezu et al., 2012)
**Figure 7**		
KGY9344-2	spa2Δ::ura4^{+} h^{+}	This study
KGY45	975 h^{+}	Lab stock
**Figure S2**		
KGY15975	spa2-GFP:kanMX6 ade6-M216 ura4-D18 leu1-32 h^{+}	Lab stock
KGY15148	spa2(1-500)-GFP:kanMX6 ade6-M21X ura4-D18 leu1-32 h^{-}	This study
KGY15201	spa2(1-360)-GFP:kanMX6 ade6-M21X ura4-D18 leu1-32 h^{-}	This study
KGY15613	spa2(1-305)-GFP:kanMX6 ade6-M21X ura4-D18 leu1-32 h^{-}	This study
KGY15754	spa2-P191A-GFP:kanMX6 ade6-M21X ura4-D18 leu1-32 h^{-}	Lab stock
KGY11996	spa2-GFP:kanMX6 pos1Δ::ura4^{+} ade6-M21X ura4-D18 leu1-32 h^{+}	This study
KGY9250-2	spa2Δ(CC1,221-301)-GFP:kanMX6 ade6-M21X ura4-D18 leu1-32 h^{+}	This study
KGY9260-2	spa2Δ(CC2,310-365)-GFP:kanMX6 ade6-M21X ura4-D18 leu1-32 h^{+}	This study
KGY9374-2	spa2Δ(FAT1,391-497)-GFP:kanMX6 ade6-M21X ura4-D18 leu1-32 h^{+}	This study
KGY15819	spa2-R58A,R59A-GFP:kanMX6 ade6-M21X ura4-D18 leu1-32 h^{-}	This study
**Figure S3**		
KGY15975	spa2-GFP:kanMX6 ade6-M216 ura4-D18 leu1-32 h^{+}	Lab stock
KGY11996	spa2-GFP:kanMX6 pos1Δ::ura4^{+} ade6-M21X ura4-D18 leu1-32 h^{+}	This study

Journal of Cell Science • Supplementary information

KGY1296	*PJ69-4A MATa trp1-190 leu2-3,112 ura3-52 his3-200 gal4 delete gal80 delete LYS2 replaced by GAL1-HIS3 GAL2-ADE2 met2 replaced by GAL-lacZ*	Lab stock
KGY15996	*spa2-GFP:kanMX6/spa2-FLAG:kanMX6 ade6-M216/ade6-M210 leu1-32/leu1-32 ura4-D18/ura4-D18 h⁻/h⁺*	This study
KGY18589	*spa2-GFP:kanMX6/spa2-FLAG:kanMX6 pos1Δ::ura4⁺/pos1Δ::ura4⁺ ade6-M216/ade6-M210 leu1-32/leu1-32 ura4-D18/ura4-D18 h⁻/h⁺*	This study
KGY6894	*spa2-FLAG:kanMX6 ade6-M21X ura4-D18 leu1-32 h⁻*	Lab stock
**Figure S4**		
KGY15975	*spa2-GFP:kanMX6 ade6-M216 ura4-D18 leu1-32 h⁺*	Lab stock
KGY10132-2	*spa2-GFP:kanMX6 mkh1Δ::ura4⁺ ade6-M21X ura4-D18 leu1-32 h⁻*	This study
KGY738-2	*mkh1-mNG:hphMX6 ade6-M21X ura4-D18 leu1-32 h⁺*	This study
KGY9702-2	*mkh1-mNG:hphMX6 spa2Δ::ura4⁺ ade6-M21X ura4-D18 leu1-32 h⁻*	This study
KGY9302-2	*pek1-linker-mNG:kanMX6 ade6-M21X ura4-D18 leu1-32 h⁺*	This study
KGY9304-2	*pek1-linker-mNG:kanMX6 spa2Δ::ura4⁺ ade6-M21X ura4-D18 leu1-32 h⁻*	This study
KGY9309-2	*spa2-GFP:kanMX6 gyp3Δ::kanMX6 ade6-M21X ura4-D18 leu1-32 h⁻*	This study
KGY9310-2	*spa2-GFP:kanMX6 bud6Δ::kanMX6 ade6-M21X ura4-D18 leu1-32 h⁻*	This study
KGY9373-2	*spa2-GFP:kanMX6 for3Δ::ura4⁺ ade6-M21X ura4-D18 leu1-32 h⁻*	This study
KGY12024	*for3-GFP$_3$:kanMX6 ade6-M21X ura4-D18 leu1-32 h⁻*	Lab stock
KGY1913-2	*for3-GFP$_3$:kanMX6 spa2Δ::ura4⁺ ade6-M21X ura4-D18 leu1-32 h⁻*	This study
KGY8906	*bud6-GFP$_3$:kanMX6 ade6-M21X ura4-D18 leu1-32 h⁺*	Lab stock
KGY9395	*bud6-GFP$_3$:kanMX6 spa2Δ::ura4⁺ ade6-M21X ura4-D18 leu1-32 h⁻*	This study
**Figure S5**		
KGY6004	*crn1-GFP:kanMX6 h⁻*	Lab stock
KGY9394	*spa2-GFP:kanMX6 exo70Δ::ura4⁺ ade6-M21X ura4-D18 leu1-32 h?*	This study
KGY10861	*spa2-GFP:kanMX6 ade6-M21X ura4-D18 leu1-32 h⁺*	This study
KGY11965	*spa2-GFP:kanMX6 orb6-167 ade6-M21X ura4-D18 leu1-32 h?*	This study
KGY15975	*spa2-GFP:kanMX6 ade6-M21X ura4-D18 leu1-32 h⁺*	Lab stock

Supplementary references

Altschul, S. F., Gish, W., Miller, W., Myers, E. W. and Lipman, D. J. (1990). Basic local alignment search tool. *J. Mol. Biol.* **215**, 403-410. doi:10.1016/S0022-2836(05)80360-2

Engel, S. R., Aleksander, S., Nash, R. S., Wong, E. D., Weng, S., Miyasato, S. R., Sherlock, G. and Cherry, J. M. (2024). *Saccharomyces* genome database: advances in genome annotation, expanded biochemical pathways, and other key enhancements. *bioRxiv* 2024.09.16.613348. doi:10.1101/2024.09.16.613348

Foltman, M., Filali-Mouncef, Y., Crespo, D. and Sanchez-Diaz, A. (2018). Cell polarity protein Spa2 coordinates Chs2 incorporation at the division site in budding yeast. *PLoS Genet.* **14**, e1007299. doi:10.1371/journal.pgen.1007299

Moreno, D., Neller, J., Kestler, H. A., Kraus, J., Dünkler, A. and Johnsson, N. (2013). A fluorescent reporter for mapping cellular protein-protein interactions in time and space. *Mol. Syst. Biol.* **9**, 647. doi:10.1038/msb.2013.3

Shih, J. L., Reck-Peterson, S. L., Newitt, R., Mooseker, M. S., Aebersold, R. and Herskowitz, I. (2005). Cell polarity protein Spa2P associates with proteins involved in actin function in Saccharomyces cerevisiae. *Mol. Biol. Cell* **16**, 4595-4608. doi:10.1091/mbc.e05-02-0108
